# A single residue controls electron transfer gating in photosynthetic reaction centers

**DOI:** 10.1038/srep44580

**Published:** 2017-03-16

**Authors:** Oksana Shlyk, Ilan Samish, Martina Matěnová, Alexander Dulebo, Helena Poláková, David Kaftan, Avigdor Scherz

**Affiliations:** 1 The Weizmann Institute of Science, Department of Plant and Environmental Sciences, 76100 Rehovot, Israel; 2 University of South Bohemia in České Budějovice, Faculty of Science, 37005 České Budějovice, Czech Republic; 3 Institute of Microbiology CAS, Department of Phototrophic Microorganisms, 37981 Trebon, Czech Republic

## Abstract

Interquinone *Q*_*A*_^−^ → *Q*_*B*_ electron-transfer (ET) in isolated photosystem II reaction centers (PSII-RC) is protein-gated. The temperature-dependent gating frequency “*k”* is described by the Eyring equation till levelling off at T ≥ 240 °K. Although central to photosynthesis, the gating mechanism has not been resolved and due to experimental limitations, could not be explored *in vivo*. Here we mimic the temperature dependency of “*k”* by enlarging *V*_*D1-208*_, the volume of a single residue at the crossing point of the D1 and D2 PSII-RC subunits in *Synechocystis* 6803 whole cells. By controlling the interactions of the D1/D2 subunits, *V*_*D1-208*_ (or 1/T) determines the frequency of attaining an ET-active conformation. Decelerated ET, impaired photosynthesis, D1 repair rate and overall cell physiology upon increasing *V*_*D1-208*_ to above 130 Å^3^, rationalize the >99% conservation of small residues at *D1-208* and its homologous motif in non-oxygenic bacteria. The experimental means and resolved mechanism are relevant for numerous transmembrane protein-gated reactions.

PSII-RCs and RCs of green nonsulfur bacteria (Chloroflexi), purple bacteria (phototrophic Proteobacteria) and the newly discovered photosynthetic Gemmatimonadetes^1^, are Type-II RCs responsible for light-induced charge separation across the photosynthetic membrane^2^. The functional core of PSII RCs comprises ten transmembrane (TM) helices of the D1 and D2 subunits, which display C2 pseudo-symmetry. The *d* and *e* helices of the two subunits form a four-helix bundle that holds the ET-facilitating cofactors (Fig. 1a). The luminal arms of these four TM helices (hereafter, denoted *d1, d2, e1, e2*) coordinate a non-heme iron and bind *Q*_*A*_ and *Q*_*B*_ on *d2* and *d1*, respectively. These components define the electron acceptor side of the RC complex. The cytosolic arm of the helices harbors the electron-donor side of the complex, including a cluster of four chlorophylls and two pheophytins^3^. The primary ET reaction comprises ultrafast, light-induced tunneling of an electron across the clustered pigments, followed by reduction of *Q*_*A*_^4^,^5^,^6^,^7^. Then, a slower ET from *Q*_*A*_ to *Q*_*B*_, coupled with protonation, follows with more extensive energy dissipation that stabilizes charge separation^8^,^9^,^10^,^11^,^12^,^13^. The apparent *Q*^*−*^_*A*_ → *Q*_*B*_ ET rate is ~3 orders of magnitude slower than that expected by tunneling (ca. ~10^6^ s^−1^) using modified Marcus equation; thus suggesting that tunneling is not the rate-limiting step in this reaction^14^,^15^. The temperature-dependent *Q*^*−*^_*A*_ → *Q*_*B*_ ET rate has been considered evidence for a protein-gated mechanism in both oxygenic^8^,^11^,^16^ and non-oxygenic^12^,^17^,^18^,^19^ Type-II RCs. The first reduction of *Q*_*B*_ by *Q*_*A*_^*−*^ involves a protein conformational change that brings the reactants from a non-active (dark) to the transient and favorable active (light) ET conformation, followed by rapid electron tunneling from *Q*_*A*_ to *Q*_*B*_, accompanied by protonation^7^,^10^,^12^,^13^. *In vitro* RCs that were cooled to cryogenic temperatures under light, continued to perform light-induced ET while those cooled in the dark did not, an observation made first in non-oxygenic Type-II RCs and termed the Kleinfeld effect^20^.

Understanding the protein dynamics that gate primary *Q*_*B*_ reduction and its dependence on the ambient temperature, remains a long-standing challenge in photosynthesis research^9^,^11^,^21^,^22^. The temperature (T) effect has typically been explained in terms of protein flexibility^4^,^5^,^11^,^16^,^18^,^23^, a property that allows the protein to undergo structural change in response to the presence of other molecules and/or variations in the environment^24^.

While flexibility in one protein region can strongly affect the local conformation attained at a remote site^24^,^25^, most queries into the origin of the ET-associated protein conformational change in Type-II RCs have focused on the quinones and their immediate vicinity. In particular, temperature increases were shown to result in elevated frequency and amplitude of individual atom displacements at and around the non-heme iron and quinone sites^4^,^16^,^26^. Decreased temperatures have been correlated with increased binding interactions in this domain, leading to less frequent displacements and consequentially reduced probability to attain the conformation favorable for ET^11^. Other experimental and theoretical studies have aimed at resolving the relative orientations, electron distribution, redox potentials, formation or elimination of hydrogen bonding to *Q*_*B*_^*−*^ and changes in the amino acids that ligate the non-heme iron^9^,^11^,^13^,^22^,^27^,^28^,^29^,^30^,^31^,^32^,^33^,^34^. However, to the best of our knowledge, none have successfully generated a model that quantitatively relates well-defined protein conformations with the ET frequency. Furthermore, none of the proposed models were evaluable in whole cells, where monitoring of photosynthetic ET and subsequent processes below the freezing point is impossible. Thus the RC gating phenomenon and its impact on whole-cell photosynthetic activity and physiology remained unresolved in oxygenic and non-oxygenic phototrophs alike.

In this study, we first set out to find a means of mimicking the temperature effect on protein conformations and the resulting *Q*_*A*_^*−*^ → *Q*_*B*_ ET rate *in vivo*, without cooling the cells. Once establishing such a model, the impact of “temperature-like” perturbations on light-induced ET, photosynthetic machinery and whole cell physiology could be quantitatively monitored.

We hypothesized that reversible thermal motion of the *Q*_*A*_ and *Q*_*B*_ binding helices (*d2* and *d1*) relative to each other “opens” and “closes” a “gate” for ET tunneling from *Q*_*A*_ to *Q*_*B*_. We further hypothesized that as in other systems undergoing “gating”^35^,^36^,^37^, a hinge controls the frequency of this movement. Following careful examination of Type-II RC structures, we speculated that the hinge is located within a GxxxG-like TM helix-helix interface motif, where G denotes small amino acid residues *V*_*res*_ < 130 Å^3^ (i.e., Gly, Ser, Cys, Thr and Ala)^38^,^39^,^40^. As a candidate we selected D1-208 within the TM G_208_xxxG_212_-like motif in the *d1* helix of PSII RC. This motif faces a G_207_xxxG_211_-like motif in the *d2* helix (Fig. 1b). Following transition state theory, residues that comprise a hinge should control the binding interactions among the *d1* and *d2* and consequently determine the ratio of ET-active versus inactive protein conformations. A similar GxxxG-like motif consisting of L-185 to L-189 in RC of non-oxygenic bacteria interfaces M-212 to M-216 at the crossing of *d1* and *d2* helices (Supplementary Fig. 1A). The presented *in silico, in vivo* and *in vitro* studies focused on the PSII RC motif, with particular emphasis on D1-208.

## Results

### Evolutionary conservation of a small residue motif at the *d1/d2* TM interface

**Figure 1:**
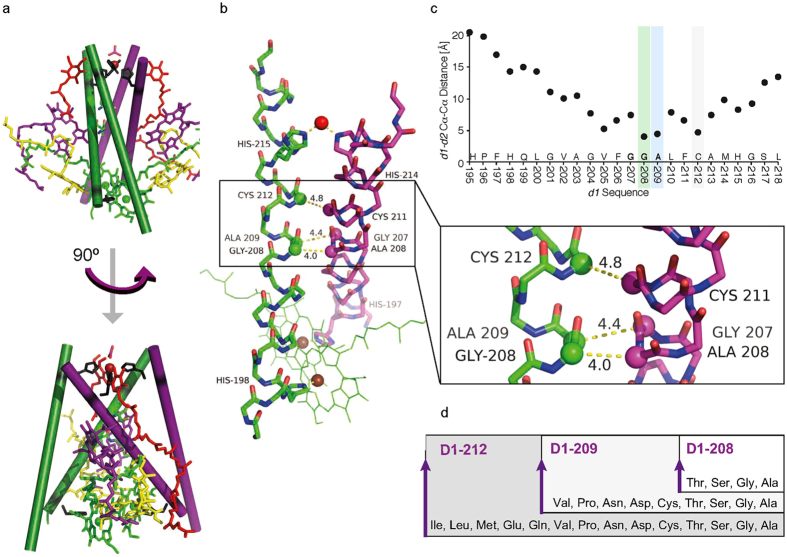
The PSII RC complex (PDB: 3wu2). (**a**) The four-helix bundle includes helices *d1, e1, d2, e2* of the D1 (green) and D2 (purple) subunits. It holds a central cluster of chlorophylls (P680), including a central pair (green) flanked by additional Chls (yellow), Phe (purple), quinones (red) and the non-heme iron (red sphere), that is further ligated by a bicarbonate (red). See Supplemental Fig. 1 for a depiction of all the 10 TM helices of subunits D1 and D2. Within the four-helix bundle, intersubunit interactions are found at the center of *d1* (D1-208 and D1-209) and *d2* helices, around the non-heme iron, where histidines (black) from the two subunits ligate an iron ion, and at the central P680 Chls, where histidines from the two subunits ligate the Mg ions. A 90^o^ turn (bottom) shows that the *d* helices form an ‘X’ shape with D1-208 at their center. (**b**) The intersubunit Cα-Cα distances between of the studied GxxxG-like motifs: D1 residues Gly208, Ala209, and Cys212 and their closest D2 residues Ala208, Gly207 and Cys211, respectively. The histidines ligating the non-heme iron (red sphere) and central P680 Chls (green) are depicted. (**c**) The intersubunit Cα-Cα distances (y-axis) between *d1* (x-axis) and the closest Cα on *d2* (see Supplementary Fig. 1, for detailed table). (**d**) The list of residues enabling photoautotrophic growth for the three studied amino acids includes 4 residues for D1-208, 9 for D1-209 and 14 for D1-212.

D1-208 and D1-212 delimit a G_208_xxxG_212_-like motif. The motif is located at the membrane’s hydrophobic core, with the C_α_ centers of the terminal residues positioned at a membrane depth of 2.46 and −6.73 Å (PDB: 3wu2, ref. 41) from the membrane center, respectively (Supplementary Table 1). These residues face a *d2* helix G_207_xxxG_211_-like motif where D1-208Gly is in close contact with D2-208Ala (4.0 Å Cα to Cα, Fig. 1c and Supplementary Fig. 1B). Such an interhelical distance is regarded as an extremely and atypically close distance^39^ that should be accompanied by strong interhelical interactions.

D1-208Gly is flanked by small residues D1-207Gly on the donor side and by D1-209Ala on the acceptor side of the PSII, forming a unique ‘consecutive-small’ residue motif. The distance between the respective *d1* and *d2* helices then increases as approaching the donor and acceptor sides of PSII (Fig. 1c). The Cα to Cα distance between residues D1-209 and D2-207Gly (acceptor side) is 4.4 Å and between D2-211Cys and the accepting D1-212 is 4.8 Å. The next interface pairs, namely D1-205Val and D2-208Ala and D1-202Val and D2-205Leu are positioned at Cα to Cα distances of 5.3 and 10.1 Å, respectively (Fig. 1c and Supplementary Fig. 1B).

Screening of all available D1 PSII RC sequences demonstrated conservation of three consecutive small residues between D1-207 and D1-209 in 4665 sequences. The vast majority (89.4%) were GGS, while the remaining (10.6%) were GGA sequences. Overall, the few cases in which the motif was not fully conserved small residues still comprised the sequence, which were often non-viable, e.g. in viruses (Supplementary Table 2).

### Small amino acid volume at the *d1/d2* TM interface is essential for photoautotrophic growth of *Synechocystis* sp. PCC 6803

Using PCR-based saturation mutagenesis in *Synechocystis* sp. PCC 6803, we aimed to experimentally assess the role of most closely situated *d1* TM sites that may be involved in intersubunit interactions with the *d2* helix (Fig. 1). All viable mutants contained functional RCs that maintained photoautotrophic growth and presented *Q*_*A*_^*—*^ → *Q*_*B*_ ET. As shown in Fig. 1d, saturation mutagenesis at the D1-212 site yielded the wild-type (*wt*) (D1-Ser212Ser) as well as thirteen photoautrophic isogenic mutant strains bearing Gly, Ala, Cys, Thr, Asn, Asp, Pro, Val, Gln, Glu, Ile, Leu, or Met at the 212 position. Mutagenesis at the D1-209 site yielded the *wt* (D1-Ser209Ser) and eight (Gly, Ala, Cys, Thr, Asn, Asp, Pro, Val) mutants (Fig. 1d). The same mutagenesis procedure at the D1-208 site yielded the *wt* (D1-Gly208Gly) and only three photoautotrophically competent mutants, bearing Ala, Ser or Thr at the 208 position (Fig. 1d), hereafter denoted D1-Gly208Ala, D1-Gly208Ser and D1-Gly208Thr. The remaining 16 possible amino acid substitutions at D1-208 failed to support photoautotrophic growth. Interestingly, bulky aromatic amino acid residues (Tyr, Trp, Phe, and His) and positively charged amino acid residues (Arg and Lys)^42^ failed to support photoautotrophic growth when substituted into these three target sites despite the fact that their codons were available. In summary, the allowed maximal residue volume decreases with decreasing distances from the crossing point of the *d1*/*d2* helices (Fig. 1).

### Volume increase of residue D1-208 and temperature reductions display a similar effect on the protein-gated ET

**Figure 2:**
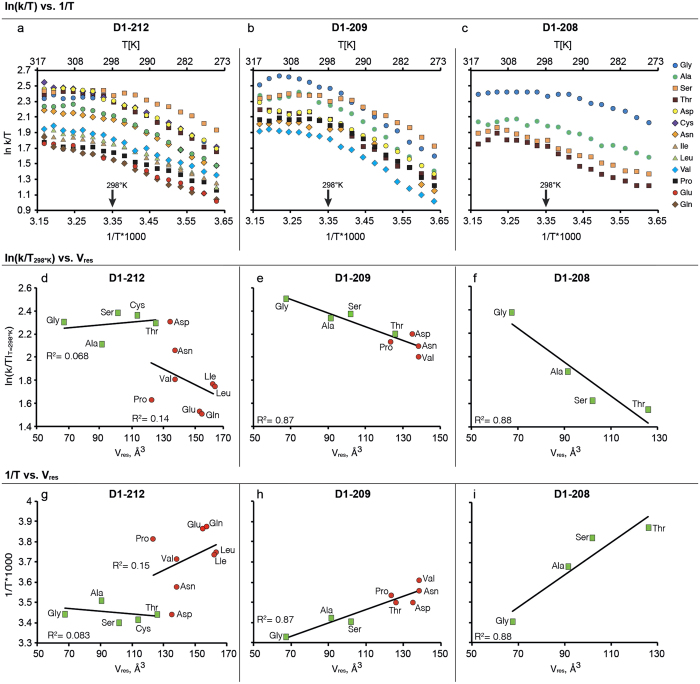
The impact of mutations in the *d1/d2* TM interface residues D1-208, D1-209 and D1-212 on k, the Q_A_^−^ → Q_B_ ET rate. (**a–c**) Ln(k/T) is presented as a function of the temperature, T. (**d–f**) The measured *Q*_*A*_^*−*^ → *Q*_*B*_ ET rate at 25 °C is presented as a function of the residue volume (*V*_*res*_) for the three sites. Utilizing the linear relationship found between Ln(k/T) and *V*_*res*_, for each of the three sites, each mutant was given a temperature that provides for the same ET rate as that of the *wt* at room temperature. (**g**–**i**) Correlation between the temperature of the “effective” cooling effect and the residue volume.

The effect of mutations at D1-208, D1-209 and D1-212 on the rate of protein conformational change, as manifested by the apparent *Q*^*−*^_*A*_ → *Q*_*B*_ ET rates (Fig. 2a–c and Supplementary Fig. 2), was followed by monitoring *in vivo* chlorophyll fluorescence decay in whole cells, at temperatures ranging from 0–50 °C. The procedures selected for the whole cell preparation assured the measurement of the first *Q*^*−*^_*A*_ → *Q*_*B*_. These include adaptation to dark; fluorescence measurements modality and deconvolution of the collected data (see online methods). The *in vivo* ET rates of *wt* and all viable D1-212, 209 and 208 mutants were derived as described^43^. Mutants D1-Ser212Met and D1-Ser209Cys, could not be stabilized during growth with significant reversal to the *wt* strain hence excluded from the analysis. The experimentally derived standard deviations of the measured *k* values are presented for the three mutated D1 residues in Supplementary Fig. 2b–d. The Ln(k/T) displays a linear correlation with 1/T for *wt Synechocystis* 6803, till leveling off at, or slightly above *T*_*0*_, the physiological temperature of the organism habitat^43^ as illustrated in Fig. 2a–c.

**Figure 3:**
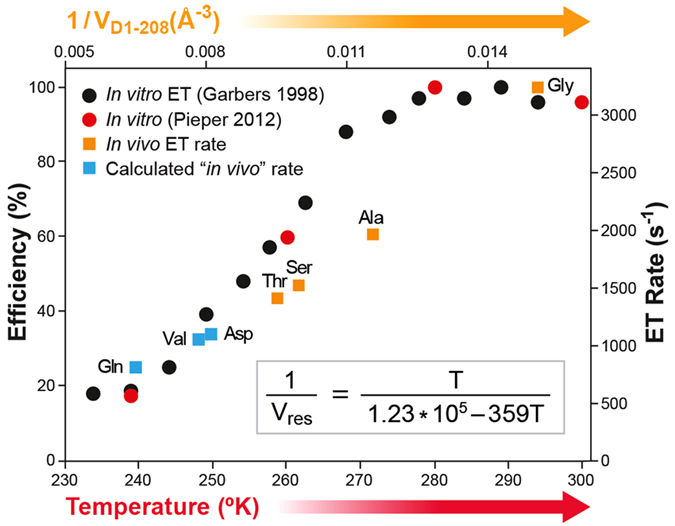
*Q*_*A*_^*−*^ → *Q*_*B*_ ET efficiency and rate in isolated PSII-RC and whole cells, as a function of temperature and D1-208 residue volume (*V*_*D1-208*_), respectively. The data for the *in vitro* ET efficiency and rate were taken from Fig. 5 in ref. 16 and Fig. 1 in ref. 4, respectively. The ET rates corresponding to particular 1/*V*_*D1-208*_ values were taken from Fig. 1 and Supplementary Fig. 3C. The alignment of 1/*V*_*D1-208*_ and T scales (X axes) is based on Equation 1.

Next, for all D1-212, D1-209, and D1-208 mutants, Ln(k/T_298°K_), i.e. the ET rate at T = 298 °K, was plotted (Fig. 2d–f) as a function of the residue volume *V*_*res*_ till reaching *T*_*0*_. as it shown before^43^. The ET rate constants for the D1-212 mutants clustered into the previously^43^ defined group I (V^I^_res_ ≤ 130 Å^3^, green data-points in Fig. 2) and group II (Pro and residues with V^II^_res_ > 130 Å^3^, red data-points in Fig. 2. The Ln(k/T_298°K_) values for group I were in the range of 2.1–2.4 (Fig. 2d), while those for group II ranged between 1.5 and 2.0. For D1-212, small changes in the packing values of group I residues were previously shown to facilitate the acclimation of mesophiles and thermophiles to their ambient temperatures^43^. For D1-209, a linear regression (Ln(k/T_298°K_) = −5.9·*V*_*D1-209*_ + 2.9, R^2^ = 0.87) was obtained for all viable mutants. The allowed maximal residue size was significantly smaller as compared to D1-212 (150 Å^3^ and 170 Å^3^, respectively). Occupation of the D1-208 site was only feasible with small, group I residues (*V*_*res*_ < 130 Å^3^); the plot of Ln(k/T_298°K_) vs *V*_*D1-208*_ (Fig. 2f) presented a steep linear regression (Ln(k/T) = −14.5·*V*_*D1-208*_ + 3.25, R^2^ = 0.88). Taken together, the effect of *V*_*res*_ on the rate of ET markedly increased as the site of mutation approached the closest *d1/d2* contact point (crossing point). At D1-208, this effect appeared equivalent to the previously reported effect of reduced PSII RC temperatures in membrane fragments or thylakoids^4^,^16^. Both interventions strongly “cool down” the rate of the protein-gated *Q*^*−*^_*A*_ → *Q*_*B*_ ET (Fig. 3).

To formulate this intriguing equivalence, we extrapolated the Ln(k/T) vs. (1/T) plot beyond the measured temperature range (dashed boxes in Supplementary Fig. 3 represent the extrapolated region) for the *wt* (gray diamonds in Supplementary Fig. 3). Then, the rate constants measured at 25 °C for the different viable mutants (k^mut^_298°K_, Fig. 2) at D1-208, D1-209 and D1-212 sites, were positioned on the extrapolated wild type plot (colored squared, Supplementary Fig. 3). Next, for each *V*_*res*_, we found a corresponding 1/T that provided the same ET rate (arrows, Supplementary Fig. 3). The derived 1/T values were used to construct a plot of 1/T vs. *V*_*res*_ for each site (Fig. 2g–i). These plots provide the “effective” cooling effect introduced by a certain residue volume at this site.


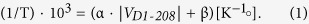


For example in the D1-208 mutants α and β equal to 0.0081 and 2.91 respectively.

Thus, as demonstrated in Fig. 2i, a D1-208 residue volume increase of 24 Å^3^ (e.g. Gly to Ala) is equivalent to an effective cooling of ∆T ~ (−14 °K). Introduction of additional 35 Å^3^ (Thr) is equivalent to an additional cooling of ∆T ~ (−22 °K). Introduction of Gln (*V*_*res*_ = 156.4 Å^3^) at D1-208 would bring down the “effective temperature” for ET to 239.5 °K (Supplementary Fig. 3C).

Previous works have shown that exposing Type-II RCs to low temperatures, freezes both *Q*^*−*^_*A*_ → *Q*_*B*_ ET and the average displacements of atoms throughout the PSII RC protein scaffold^4^,^16^. The impact of T reduction, measured for isolated PSII RCs^4^,^16^, and *V*_*res*_ elevation, measured here in whole cells, on *Q*_*A*_ → *Q*_*B*_ ET were highly similar (Fig. 3 and Supplementary Table 3), strongly suggests that the D1-208 residue controls the frequency of protein shifting from an ET-inactive to an ET-active conformation.

D1-209 mutants also displayed a linear regression relationship between 1/T and *V*_*D1-209*_, albeit, more moderate when compared to D1-208 (Fig. 2h). For example, a volume increase of 24 Å^3^ was equivalent to a temperature decrease of ~8 °K (Supplementary Fig. 3B). Finally, as shown in Fig. 2g, no systematic “cooling effect” was demonstrated for increased *V*_*res*_ at D1-212. Nevertheless, as we previously showed, changing the residue packing value modified the entropy of activation and thereby the ET rate^43^. The apparent “cooling” of the *Q*^*−*^_*A*_ → *Q*_*B*_ ET with increasing *V*_*res*_ values, can be translated to increased activation free energy (*∆G*^*‡*^), as shown for D1-208, as well as for D1-209, although at a lower rate and with a somewhat lower correlation (Supplementary Fig. 4).

Detailed analysis of the enthalpic (*∆H*^*‡*^) and entropic (*∆S*^*‡*^) components of the activation free energy for each of the D1-208 mutants (Supplementary Table 4) indicated that the residue size mainly affected the entropy of activation (by up to ~3.7–4.8 kJ·mol^−1^), which was in agreement with the concept of local cooling and a previous study of the *Q*^*−*^_*A*_ → *Q*_*B*_ ET^17^. The ability to locally and systematically “cool down” the ET processes *in vivo* while leaving the rest of the cell at a physiological temperature, provides a new means of studying the physiological significance of *Q*^*−*^_*A*_ → *Q*_*B*_ ET rates in the context of whole cells.

### D1-208 volume affects whole cell growth, RC pigment composition and RC integrity

**Figure 4:**
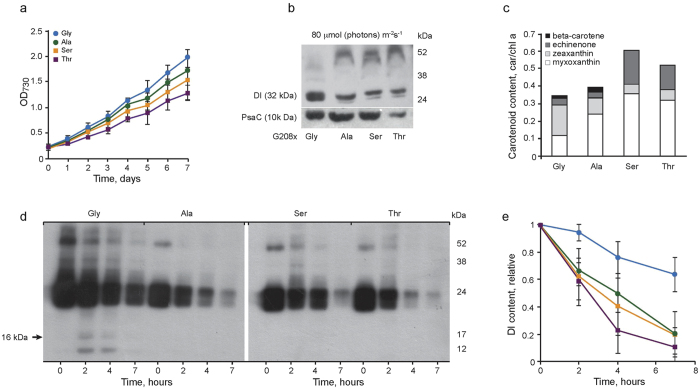
Growth rate, and pigment and protein levels in cell cultures of *wt* and D1-208 mutated *Synechocystis* sp. PCC6803. (**a**) Cell growth in the liquid BG-11 medium under standard (80 μmol (photons) m^−2^s^−1^) light intensity. Means and standard deviations are shown (n = 3). (**b**) Immunoblots of the D1 and PsaC proteins in cells grown under standard light intensity. (**c**) β-carotene, echinenone, zeaxanthin and myxoxanthophyl content in D1-208 mutant cells grown under the same light intensity; levels were normalized to the chlorophyll a concentration in the assayed sample. (**d**) Immunoblots showing changes in the D1 protein content and its degradation products during 7 h illumination at high light intensity (500 μmol (photons) m^−2^s^−1^) of the D1-208 mutants in the presence of lincomycin. Blots were immunoreacted with antibodies raised against the D1protein C-terminal sequence. (**e**) D1 protein content in *wt* and D1-208 mutants decreased during 7 hours of high light intensity exposure in the presence of lincomycin. Means and standard deviations are shown (n = 3). Color code is as in panel a.

The photoautotrophic growth rate was found to be the highest in the *wt* (Fig. 4a), which exhibited a doubling time of 0.8 days, while that of D1-Gly208Thr was 20% longer. Growth rate of the D1-Gly208Ala and D1-Gly208Ser mutants was 0.9 days. Under light conditions of 80 μmol (photons)·m^−2^·s^−1^, the D1-Gly208Ala mutant demonstrated a 50% decrease of D1 content, while D1-Gly208Ser and D1-Gly208Thr showed 60% and 70% reduction, respectively compared with the *wt* (Fig. 4b). Furthermore, the mutants exhibited larger amounts of the ~60 kDa cross-linked D1/D2 heterodimer products, relative to the *wt*. The steady state levels of the PsaC protein were equally high in the *wt*, D1-Gly208Ala and D1-Gly208Ser mutants, but were approximately 50% lower in the D1-Gly208Thr mutant (Fig. 4b). Xanthophyll carotenoids myxoxanthophyll^44^ (Pearson’s correlation R = 0.854) and echinenone (Pearson’s correlation R = 0.643) levels increased with the size of the D1-208 residue (Fig. 4c), which paralleled a significant decrease in the content of zeaxanthin (Pearson’s correlation R = −0.895) and β-carotene (Pearson’s correlation R = −0.754).

Accumulation of D1 degradation products was demonstrated in time-resolved D1 protein immunoblots of *wt* and mutated cells exposed to high light (500 μmol (photons)·m^−2^·s^−1^) in the presence of the protein synthesis inhibitor, lincomycin (Fig. 4d,e). Specifically, the 16 kDa fragment of the C-terminus was detected in the *wt* but not in the mutants (Fig. 4b). D1 degradation (Fig. 4e) appeared almost five times faster in the D1-Gly208Thr mutant compared to the *wt* and three-fold faster in D1-Gly208Ala and D1-Gly208Ser as compared to *wt*.

### Increased D1-208 volume affects *d1/d2* binding interactions

**Figure 5:**
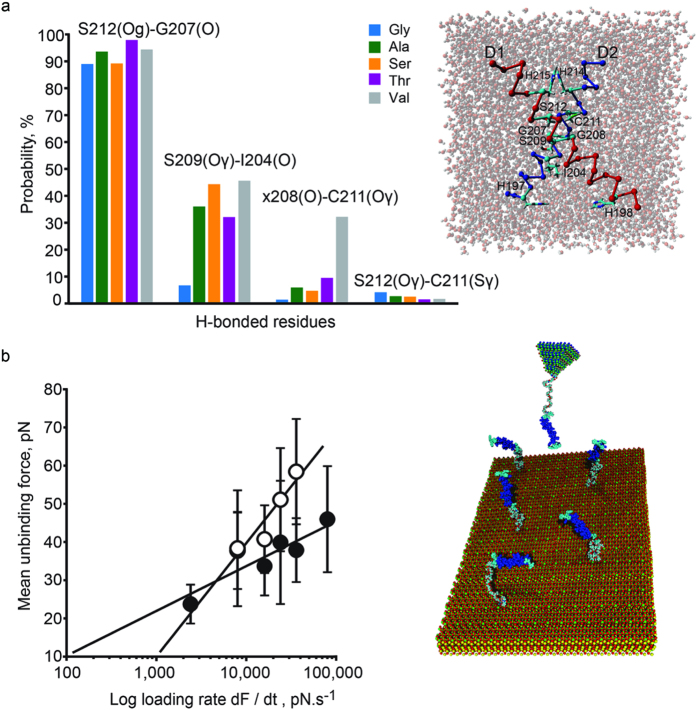
Interactions between synthetic *d* helices of the D1 and D2 subunits. (**a**) Probability for the formation of interhelical hydrogen bonds between the annotated residues of D1and D2 in D1-208 mutants (H-bond energies and distances are given in Supplementary Table 5; color codes annotate the residue occupying site D1-208). Insert: Structure of *d* helices exposed to the MD showing the aqueous environment of the simulation cell. (**b**) The mean force for unbinding the synthetic *d1*-*d2* dimer, as measured by AFM under increasing force load, illustrated for structures containing small (Gly, open circles) and relatively large (Val, closed circles) amino acids at the D1-208 site. The vertical error bars represent the standard deviation calculated from the histograms of unbinding forces binned by 5 pN (see also Supplemental Fig. 4). Insert: The receptor peptide (*d2*, down) was immobilized to a mica surface via a flexible PEG linker, with *d1* attached to the AFM tip.

To resolve the mechanism by which D1-208 *V*_*res*_ affects the thermodynamics and kinetics of *Q*^*−*^_*A*_ → *Q*_*B*_ ET, we explored the freedom of motion and binding interactions between the *d1* and *d2* helices mutated at D1-208 using molecular dynamics (MD) simulations. The probability of specific intersubunit interactions is displayed in Fig. 5a and Supplementary Table 5. The *d1/d2* trajectories depict four interhelical H-bonds with a dominant H-bond (90% probability) between *d1-*212Ser(Oγ) and *d2*-207Gly(O) (d_H_ = 1.8–1.9 Å, E = 22.5–23.7 kJ.mol^−1^, where d_H_ denotes the H-bond length). The probability of H-bond formation between *d1*-209Ser(Oγ) and *d2*-204Ile(O) increased from 7% in the *d1wt* (d = 2.0 ± 0.2 Å, E = 17.8 ± 5.7 kJ.mol^−1^) to 32 and 46% for *d1-*208Thr and *d1-208*Val, with E = 20.7 and 21.5 kJ.mol^−1^, respectively (d_H_ = 1.9 Å in both). The least probable interhelical H-bond formation in all D1-208 mutants (4% for *wt*) was between *d2*-211Cys(Sγ) and *d1-*212Ser or *d1*-212Ser(Oγ), which had projected binding energies of 11.5 and 15.7 kJ.mol^−1^, respectively.

### Increased D1-208 volume stabilizes the *d1/d2* binding

The MD simulations unveiled the impact of *V*_*res*_ values on the probability of making intersubunit H-bonding between *d1* and *d2*. In agreement with the local “cooling effect”, increased *V*_*res*_ may also strengthen the van der Waals interactions between the two helices, e.g. dipolar interaction. To test this hypothesis, AFM was applied to measure the forces required to unbind synthetic *d1* and *d2* TM analogues comprising *wt* and mutated amino acid sequences (Fig. 5b, Supplementary Fig. 5). When the D1-208 site was occupied with Gly (*wt*), Ser (*d1*-208Ser) or Val (*d1*-208Val), representing group I (Gly, Ser) and group II (Val), residues, the mean unbinding forces for the *d1wt*/*d2wt* complex decayed markedly faster with lowered loading rates, as compared to the *d1*-208Val/*d2wt* pair (Fig. 5b). Specifically, the rate of dissociation of the *d1wt/d2wt* complex at equilibrium was twice as fast as that of the *d1*-208Val*/d2wt* complex. The dissociation rate of the *d1*-208Ser/*d2wt* complex was intermediate. When normalizing and analyzing the data according to Dudko^45^, the extracted lifetimes for the *d1wt*/*d2wt, d1*-208Ser/*d2wt* and *d1*-208Val/*d2wt*, were 9, 23 and 52 ms, respectively.

## Discussion

The long-unresolved protein gating mechanism of *Q*_*A*_^*−*^ → *Q*_*B*_ ET in Type II reaction centers has been suggested to reflect specific changes in binding interaction at the quinones’ vicinity^46^. While recent serial time resolved crystallography of the PSII-RC complex appear to identify such changes, more recent study has challenged these findings^47^.

Unlike this and earlier studies, we did not attempt to solve the molecular details of the ET active and inactive conformations, e.g., the “light” and “dark” conformations^30^. Rather, we searched for specific residues in protein-protein interface region that control the frequency of shifting from one conformation to another, expecting that such information can be utilized in subsequent studies to further characterize these conformations. With this approach, we searched for RC structural elements that determine the protein flexibility and adaptability to structural changes as reflected in the frequency (rate) of the apparent *Q*_*A*_^*−*^ → *Q*_*B*_ ET. Overall, the similar dependency of RC flexibility and *Q*_*A*_^*−*^ → *Q*_*B*_ ET rates on temperature has already been recognized two decades ago^16^. Both were shown to freeze at a similar temperature: ~250 and ~200 °K for Type-II RC for oxygenic and non-oxygenic bacteria, respectively^4^. However, assignment of specific residue as the conformational gating regulators remained highly speculative, since the ambient temperature affects movement of multiple residues in protein-level experiments *in vitro*, let alone in organism-level experiments *in vivo*^9^. Nevertheless, the concept of residue and cofactor displacement at temperatures exceeding the protein freezing point, provided preliminary evidence of protein-gated ET in Type-II RC.

Our results suggest that the temperature dependence of the ET rate in PSII RC within whole cells can be mimicked under 298 K, which is the physiological temperature of the organism, by changing the *V*_*res*_ at D1-208, a single site at the *d1/d2* crossing point located within the center of the helix-helix interface. In agreement with our working hypotheses, increasing *V*_*D1-208*_ linearly increased the energy barrier for *d1/d2* unbinding and logarithmically decreased the ET rate constant. Indeed, the previously reported *in vitro* cooling effect on ET efficiency^4^,^16^ coincides with our *in vivo* findings regarding ET rate attenuation due to *V*_*res*_ changes at the D1-208 site (Fig. 3). Thus, D1-208 functions as a hinge controlling the frequency of thermal *d1 and d2* fluctuations relative to each other.

Notably, the D1-208 site is not proximal to the *Q*_*A*_/*Q*_*B*_ redox pair, yet, when examining the Type-II RC structures, the effect of such a remote site is not surprising. The *d* helices in both oxygenic and non-oxygenic organisms are non-covalently bound at the (Fig. 1): (a) non-heme iron, ligated by histidines of the two *d* helices, the same residues that also bind *Q*_*A*_ and *Q*_*B*_, (b) *d1/d2* central TM crossing interface where inter-helical H-bonds and hydrophobic interactions tie the helices to each other, and (c) central (B)Chls cluster^3^,^11^,^26^. Following this emerging insight of the *d1* to *d2* binding regions, interactions at the *d1*/*d2* crossing-interface are expected to markedly impact the freedom of movement (flexibility) of these helices at their quinone binding residues. Similar remote effects on protein flexibility and functionality were previously reported for numerous protein systems^24^,^25^. The known sensitivity of the non-heme iron coordination and spin state to the collective motions of the RC protein core are in line with such a “remote” effect on protein flexibility^5^,^26^,^48^,^49^.

The transition state that enables ET is expected to be temporarily stable. This may be facilitated by an H-bond network re-arrangement along the GxxxG-like motif, such as exchange of the H-bond D1208Gly^…^D2-211Cys for D1-212Ser^….^D1-271Met, which decreases the probability (lower entropy) of escape from the transition state^17^,^50^. Indeed, the entropy calculations show a ~3.7–4.8 kJmol^−1^ energy barrier between *wt* (Gly) and Ser and Thr mutants at the D1-208 site (Supplementary Table 4).

The importance of *d1/d2* thermal fluctuations at the D1-208 site was further corroborated by MD analysis. According to our results (Fig. 5a), the wild type Gly at the D1-208 site does not participate in interhelical H-bonding. In contrast, Thr and even Val (as example of Class II, high-*V*_*res*_, residues) exhibited a nearly persistent H-bond (30% materialization). Moreover, the AFM analysis of *d1*/*d2* TM peptides showed that the increased lifetimes of the paired peptides corresponded with increased stability of helix-helix binding, which positively correlated with D1-208 size (Fig. 5b). It also provided a metric of protein flexibility, estimated by the frequency of shifting from one *d1/d2* conformation to another, manifested by unbinding and temporal rebinding at and around the D1-208 site. In contrast to most GxxxG-like motifs at TM helix interfaces, typically flanked by bulky β-branched residues that rigidify the protein contact domain^39^,^40^, D1-208 is flanked by conserved small residues, which extend the flexibility of this region (Supplementary Fig. 1A).

**Figure 6:**
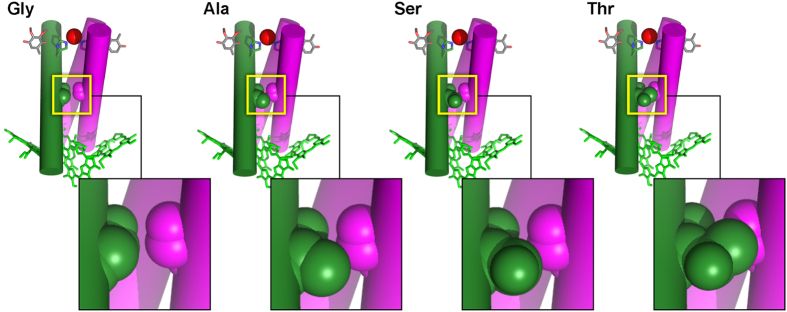
Increased *V*_*res*_ at D1-208 increases the interaction between the *d1* and *d2* helices. Using space filling models for different amino-acids at the D1-208 locus, the increased contact interaction between the two helices is depicted. See Supplementary Table 3 for the precise volume of D1-208 in the different mutants.

Figure 6 and Supplementary Fig. 6 illustrate the proposed mechanism for protein-gated ET in Type II RC, which follows the transition state theory Eyring equation (equation (2)).


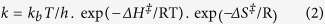


Binding interactions between the *d1* and *d2* helices at the crossing point (e.g., D1-208 in PSII RCs) preserve the Type II RC in an ET-inactive conformation. The collective motions of the protein scaffold in the dark below or at the physiological temperature, T, are insufficient to unbind the two helices. The additional energy required to release the binding, i.e., to “open” the gate, and achieve the ET active *d1/d2* geometry, is provided by the exothermic ET from the (bacterio)chlorophyll cluster to *Q*_*A*_ (dissipation of ~6 kCal/mol^51^. An increased 1/T reduces the value of the first exponent in equation (2) and thereby the value of *“k”*. Increased *V*_*D1-208*_ increases the negative value of *ΔS*^*‡*^(Supplementary Table 1), resulting in a decreased value of the second exponent. Hence, increased *V*_*D1-208*_ is equivalent to increased 1/T, accounting for the equality presented in equation (1).

This mechanism provides a straightforward rationale for the conservation of small residues at the *d1/d2* crossing of Type-II RC (Fig. 6). Such residues provide a hinge that maximizes the frequency of helices displacement relative to each other at physiological temperatures. Larger residues would markedly reduce (Fig. 6) or “cool down” the unbinding displacement frequency of *d1* versus *d2,* and thereby, the frequency of *Q*^*−*^_*A*_ to *Q*_*B*_ ET. Moreover, slow gating may allow for charge recombination, generation of toxic reactive oxygen species and rapid degradation of the photosynthetic machinery, particularly, at high light intensities.

The ability to mimic the temperature effect at cryogenic temperatures by a single site modification, allowed us to isolate the *in vivo* impact of the conformation-gated ET frequency from unrelated enzymatic reactions, such as autotrophic growth rates, D1 and psaC protein content, carotenoid profiling, and PSII RC degradation (Fig. 4). For example, we showed that attenuation of the ET rate *in vivo*, following enhanced *d1/d2* binding (introduced by D1-Gly208Thr), resulted in significant modification of the carotenoid profile. It was also found to facilitate cross-linking of the D1 and D2 protein subunits, thereby attenuating D1 repair (Fig. 4b). Similar crosslinking was previously observed under high-intensity light stress condition, where *Q*_*A*_ reduction did not match *Q*_*A*_^*−*^ oxidation^52^.

The amino-acid sequences at the TM interface of the *d1* and *d2* helices from the L and M subunits in non-oxygenic bacteria (Supplementary Fig. 1) suggest a similar mechanism of conformation-gated ET. This includes similar intersubunit hydrogen-bonding patterns^17^ and rare consecutive small residues within a GxxxG-like motif (Supplementary Fig. 1). Importantly, the distance between the *d1* and *d2* helices at the crossing point of the L and M protein subunits is somewhat larger than in PSII RC (Supplementary Fig. 1B). Thus, group I residues larger than Gly (e.g. Ala) may provide a better hinge for the conformational change of the non-oxygenic RC, in agreement with the observed sequence (Supplementary Fig. 1).

## Methods

### Sequence conservation

A BLAST sequence search^53^ of the D1 PSII subunit was conducted allowing for 10,000 output sequences. All PSII RC resulting sequences were analyzed. These displayed an expectation value (e-value) of better than 9 × 10^−148^ as well as 82% identity over 243 residues. This e-value was chosen as below it bacterial reaction centers were present in the BLAST results.

### Photosystem II structural analysis

The PSII structure from *T. vulcanus* at a resolution of 1.9 Å (PDB code 3wu2) was studied as the highest-resolution available structure^41^. To validate the findings, other structures were analyzed including from *T. vulcanus* (PDB code 4il6^54^ with 2.1 Å-resolution) and from *T. elongatus* (PDB codes 4pj0^55^, 3bz1^56^, and 2axt^57^ displaying 2.44, 2.9 and 3.0 Å-resolution, respectively).

Alignment of the protein complexes relative to the membrane such that the z-coordinate of each atoms correlates with membrane depth, was conducted using the OPM^58^ or Ez^59^ servers. Hydrogens were added to the protein complex using REDUCE^60^. Rotamers were scanned using SCWRL^61^. Correlation of Gibbs activation energy to the volume of the corresponding residues was conducted using the residue volume scale of Wodak^42^.

### Preparation of *Synechocystis* 6803 mutants

The D1-208, D1-209 and D1-212 mutant libraries were constructed using *wt Synechocystis* 6803 that contained intact *psb*AII gene with kanamycin resistance cassette introduced downstream of the *psb*AII at the StuI site. Two other gene copies *psb*AI and *psb*AIII were inactivated by spectinomycin and chloramphenicol resistance cassettes respectively^62^.

PCR-based saturation mutagenesis of *Synechocystis* 6803 *psb*AII gene that codes for the D1 protein was carried out as described (Supplementary Fig. 7 and Supplementary Table 6). The 3′ fragment of the *psb*AII gene carrying the mutation at positions 622-624 corresponding to the D1-208^63^ was made with P3 primer and the degenerated primer P208 coding for all amino acids in total. Similarly, the *psb*AII gene mutation at positions 625-627, corresponding to the D1-209 was introduced with the P3 and a degenerated primer P209. Additionally, the silent mutation was introduced in the third codon downstream the 208 or 209 mutation to create the NsiI restriction site used for screening of the transformant colonies. Primers P5and A208 for D1-208 library and P5 and A209 for D1-209 library were used to create the 5′ fragment of the gene required for homologous double recombination. Both the 5′ and the 3′ fragments were purified from agarose gel and fused in the final PCR to construct linear DNA product that was used to transform the recipient strain. The presence of the mutation was verified by restriction of the PCR product with the *NsiI* and by sequencing using P260 and P4 primers in both strands of the gene. Specific X208 and X209 oligonucleotides were designed to introduce codons in the D1-208 or D1-209 site, respectively corresponding to amino acids that were not obtained with the help of degenerated P208 or P209 primers (Supplementary Table 5). Construction of the mutant library at the D1-212 site was as described^43^.

### ET *in vivo* measurements

Cells re-suspended in fresh BG-11 medium were pre-incubated in darkness on ice for 30 minutes to completely reoxidize all PSII-RCs. Chlorophyll fluorescence decay following a single-turnover saturating flash (15 μs, ~0.1 mol photon m^−2^ s^−1^) was measured by a series of short, weak measuring flashes (pulse duration 4 μs), in the range of 0–50 ± 0.1 °C using FL-100 double-modulation fluorometer equipped with TR 2000 thermoregulator (PSI Ltd., Czech Republic) as described^43^,^50^. The normalized fluorescence was fitted to a three exponential decay function, using nonlinear least-squares fitting algorithm in MATLAB. The highest rate constant was assigned to the *Q*_*A*_^*−*^ → *Q*_*B*_ ET^64^,^65^. The ET rates in all mutants follow an Eyring exponential dependence on the temperature inverse. An example of the ET rate measured by chlorophyll fluorescence decay is provided in Supplementary Fig. 2.

### Growth conditions

Strains of *Synechocystis* 6803 were grown in 250 ml Erlenmeyer flasks containing BG-11 medium^66^ under continuous light illumination of 80 μmol (photons)·m^−2^·s^−1^ provided by Fluora^TM^ fluorescent tubes (Osram GmbH, Germany). Photoinhibitory conditions were elicited by halogen lamp (500 W) delivering illumination of 500 μmol (photons)·m^−2^·s^−1^. The cultures were kept at 30 °C in an incubator, aeration was provided by magnetic stirring. Growth rates were estimated from changes in cell density following light attenuation at 730 nm (OD_730_) and chlorophyll *a* concentration every 24 hours for 7 days. New cultures were inoculated from a starter culture and brought to an equal cell density (OD_730_ of 0.2). For the physiological measurements, cells were harvested at a mid-exponential growth phase (OD_730_ of 0.8–1) they were pelleted and re-suspended in a fresh BG-11 medium to reach 4 μM·concentration of chlorophyll *a*.

### Pigment analysis and absorption spectroscopy

Chlorophyll *a*, β-carotene and xanthophylls (myxoxanthophyll, zeaxanthin, echinenone) extracted from the cells of the *wt* and the mutant strains grown at 80 μmol (photons)·m^−2^·s^−1^ light intensity were separated and identified by HPLC analysis (Fig. 4c). The Philips PU 4100 HPLC included a Spherisorb ODS-1 reversed phase column (Waters, USA) according to the procedure described^67^. Chlorophyll *a* concentration was also determined spectroscopically using Jasco V-570 spectrophotometer (Jasco Inc., USA) from cells’ methanol extract using extinction coefficient of Lichtentaller^68^.

### SDS-PAGE and immunoblotting

Before extraction of thylakoid membranes cells of all strains were brought to OD_730_ = 1. Thylakoids were isolated as described^69^, chlorophyll was measured in membrane fraction, and protein extracts of all the strains were brought to total chlorophyll content 2.5 μg before loading. Thylakoid proteins were solubilized in sodium dodecyl sulfate (SDS) buffer (0.5 M Tris-HCl pH 6.8, 1% SDS, 24% glycerol and 4% 2-mercaptoethanol) at room temperature for 1 hour, and separated by 12.5% SDS-PAGE. Separated proteins were transferred onto a polyvinylidene difluoride (PVDF) membrane (Hybond-P, Amersham). The immuno detection was performed with primary antibodies against global D1, D1 C-terminal and PsaC proteins (Agrisera, Sweden). Secondary HRP-conjugated antisera (Promega and Sigma) were visualized using enhanced chemoluminescence system (Pierce). The quantification of the amount of D1 and PsaC proteins was done by integrating variable pixel intensities with ImageJ and comparing them to a dilution series of samples.

### Molecular dynamics simulations

The 3D structures of the *D* helices in the D1 and D2 PSII subunits were obtained by *in silico* mutagenesis of PSII-RC structures (PDB: 3BZ1). The models of the *D* helices (D1-P196:221, D2-P195:N219) were placed in periodic boundary simulation boxes that were 1 nm larger than the peptides using YASARA (YASARA Biosciences GmbH, Vienna, Austria). To mimic the native structure of the *Synechocystis* 6803 *in silico* mutations were done as described^50^ at D1-209Ala and D1-212Cys sites into Ser, and D2-204Val into Ile. In addition, two lysines were added to both termini to imitate the sequence of the peptides synthesized for the dynamic force spectroscopy

*d1wt*: KKPFHMLGVAGVFGGSLFSAMHGSLVTSKK

*d2wt*: KKPFHMMGVAGILGGALLCAIHGATVENKK.

The *wt* structure of the *d1* helix, *d1wt* was subsequently mutated at the D1-208Gly site into Ala, Ser, Thr and Val yielding four mutated structures:

*d1*-208Ala: KKPFHMLGVAGVFGASLFSAMHGSLVTSKK,

*d1*-208Ser: KKPFHMLGVAGVFGSSLFSAMHGSLVTSKK,

*d1*-208Thr: KKPFHMLGVAGVFGTSLFSAMHGSLVTSKK

*d1*-208Val: KKPFHMLGVAGVFGVSLFSAMHGSLVTSKK.

Hydrogens were added according to basic chemistry rules in pH = 8.0, the boxes were filled with TIP3P water model, and sodium atoms were iteratively placed at the coordinates with the lowest electrostatic potential until the cell was neutral. Molecular dynamics simulations were run with YASARA Structure (version 11.1.19), using a time step of 1.25 fs for intra-molecular and 2.5 fs for intermolecular forces. To remove energy hot-spots and correct the covalent geometry, the structures were first energy-minimized with the Yamber3 force field^70^, using an 8.0 Å force cut off and the Particle Mesh Ewald algorithm^71^. After removal of conformational stress by a short steepest descent minimization, the procedure was continued by simulated annealing (time step 2 fs, atom velocities scaled down by 0.9 every 10th step) until convergence was reached, i.e., the energy improved by less than 50 J·mol^−1^ during 200 steps. The simulations were then run at 300 K for 20 ns at a constant pressure (NPT) to account for volume changes due to fluctuations of peptides in the solution. Graphics were created with YASARA and POVRay (www.povray.org). The probability of interhelical H-bond formation was calculated as the ratio of the number of simulated snapshots featuring the bonded state over the total count of all simulated snapshots.

### AFM and Dynamic Force Spectroscopy

Peptides corresponding to *d1* and *d2* were synthesized chemically using standard Fmoc-protected amino acids as described^72^. Silicon nitride probes MSCT of 40 pN.nm^−1^ nominal spring constant (Bruker Inc., Camarillo, USA) and freshly cleaved sheets of muscovite mica were modified with ethanolamine in order to generate free -NH groups for coupling to the ~6 nm long flexible acetal-PEG-NHS linker as described^73^. Acetal function was cleaved with 1% citric acid in water and probes (mica sheets) with the cross-linker were then immersed in the aqueous solution of the peptide. The receptor peptides *d2* (D-helices of the D2 protein) were immobilized to the mica surface via flexible PEG linker and the *d1* peptides were attached to the AFM tip. Unreacted functional groups were passivated with aqueous solution of 1 M ethanolamine. Functionalized probes and mica sheets were then washed in the 0.1% SDS and stored at 4 °C for immediate use.

The interactions between the *d1wt, d1*-208Ser, *d1*-208Val peptides and the *d2wt* peptide were investigated by measurements of force distance cycles using the atomic force microscope PicoSPM (Agilent, Tempe, AZ). Measurements were carried out in a liquid cell enclosing 0.5 mL of 0.1% SDS at room temperature. The 0.1% SDS solution was optimized for retaining at least 90% helicity of the peptides as reflected by circular dichroism measurements (in the range of 180–320 nm) using Jasco J-715 spectropolarimeter (Supplementary Fig. 8).

The sweep amplitude of the force-distance cycles was 100 nm, with the sweep frequencies ranging from 0.3 to 10 Hz. Representative retrace data of the force distance cycles display unique unbinding events as shown in Supplementary Fig. 9. Two thousand force-distance cycles were performed with each of three individually functionalized tips. To measure the unbinding forces, spring constants of cantilevers were calibrated using thermal noise method^74^. Force spectroscopy curves were analyzed according to the formalism of Baumgarten *et al*.^75^ utilizing a custom made MatLab™ script^76^ (MathWorks Inc., Natick, MA).

The probability of unbinding was ≤10% in all examined pairs of peptides. Extrapolation of the mean unbinding forces to a zero applied force was carried out using the phenomenological theory of Bell^77^. Application of Dudko’s theory allowed to extract detailed information about the thermodynamics and microscopic properties of the dissociating complex from the unbinding force data^45^ assuming that the complex dissociation proceeds through an escape from a one-dimensional harmonic potential well to rupture through a transition state.

Control measurements were performed with the *d2* peptide (immobilized on the mica surface) blocked by an excess of the respective *d1* peptide partner (Supplementary Fig. 5). Force-distance cycles measured in decane (Supplementary Fig. 5) performed at hydrophobic conditions akin to the interior of membrane yielded unbinding forces between *d1* and *d2* peptides indistinguishable from those measured in aqueous 0.1% SDS solution.

### Statistical analysis

Data are presented as mean ± standard deviation. Statistical comparisons were performed with the two-sample t-test. A value of P < 0.05 was considered statistically significant.

## Additional Information

**How to cite this article**: Shlyk, O. *et al*. A single residue controls electron transfer gating in photosynthetic reaction centers. *Sci. Rep.* **7**, 44580; doi: 10.1038/srep44580 (2017).

**Publisher's note:** Springer Nature remains neutral with regard to jurisdictional claims in published maps and institutional affiliations.

## Supplementary Material

Supplementary Dataset
