# Supplementary material for: A single residue controls electron transfer gating in photosynthetic reaction centers
Source: Sci Rep. 2017 Mar 16;7:44580. doi: 10.1038/srep44580 (PMC5353731; doi:10.1038/srep44580)
Supplement: Supplementary Dataset [file srep44580-s1.doc]

**Supplementary Data**

**A single residue controls electron transfer gating in photosynthetic reaction centers**

Oksana Shlyk^1*^, Ilan Samish^1*^, Martina Matěnová ^2^, Alexander Dulebo^2^, Helena Poláková^2^, David Kaftan^2,3*^ and Avigdor Scherz^1*^

^1^ The Weizmann Institute of Science, Department of Plant and Environmental Sciences, 76100 Rehovot, Israel

^2^ University of South Bohemia in České Budějovice, Faculty of Science, 37005 České Budějovice, Czech Republic

^3^ Institute of Microbiology CAS, Department of Phototrophic Microorganisms, 37981 Trebon, Czech Republic

^*^These authors contributed equally to this work. Correspondence and requests for materials should be addressed to A.S. (email: [Avigdor.scherz@weizmann.ac.il](mailto:Avigdor.scherz@weizmann.ac.il)) or to D.K. (email: [david.kaftan@prf.jcu.cz](mailto:david.kaftan@prf.jcu.cz))

**Table of contents:**

| **Table/ Figure** | **Title** | **Page** |
| --- | --- | --- |
| **Abbreviations** | List of abbreviations | **3** |
| **Table 1** | The cross-membrane depth of the investigated PSII RC residues. | **4** |
| **Table 2** | List of genes in which the D1-207-209 sequence motif is not evolutionary conserved. | **5** |
| **Table 3** | The percentage of successful *Q_A_^-^*🡪*Q_B_* ET and the ET rate as a function of the ambient temperature and *V_res_* at D1-208. | **6** |
| **Table 4** | Kinetic and thermodynamics parameters of the D1-Gly208 *wt* and the three viable mutants derived. | **7** |
| **Table 5** | Hydrogen bonds formed between d helices of the D1 and D2 protein subunits during the 20 ns MD simulation. | **8** |
| **Table 6** | Oligonucleotides used in combinatorial and site-directed mutagenesis. | **9** |
| **Fig. 1** | Sequence and structure alignment of Type-II RC *d* helices. | **10** |
| **Fig. 2** | *In vivo* ET measured by chlorophyll fluorescence decay following single turnover saturating flash. | **11** |
| **Fig. 3** | Translation of *V_res_* at the D1-208, D1-209 and D1-212 positions into an effective temperature for the *Q^^_A_🡪Q_B_* ET | **12** |
| **Fig. 4** | The Gibbs activation energy DG^‡^ plotted against V_res_ for the mutations in the three studied loci. | **13** |
| **Fig. 5** | Dynamic force spectroscopy of synthetic d1/d2 interactions. | **14** |
| **Fig. 6** | Proposed mechanism for protein-gated ET in Type II RC. | **15** |
| **Fig. 7** | Two-step PCR mutagenesis procedure to introduce a specific sequence in the KS genomic DNA. | **16** |
| **Fig. 8** | Circular dichroism spectrum of synthesized peptide *d1wt.* | **17** |
| **Fig. 9** | Raw retrace data of the force distance cycle | **18** |
|  | References | **19** |

**List of abbreviations**

Å ånström, 0.1 nm

AFM atomic force microscope

C_α_ backbone carbon of amino acid

Chl chlorophyll

D1 D1 protein subunit, product of *psbA* gene

*d1* 4^th^ transmembrane helix of D1 protein

D2 D2 protein subunit, product of *psbD* gene

*d2* 4^th^ transmembrane helix of D2 protein

*d_h_* length of hydrogen bond

*e1* 5^th^ transmembrane helix *e* of D1 protein

*e2* 5^th^ transmembrane helix *e* of D2 protein

E energy

ET electron transfer

ΔG^‡^ activation free energy

ΔH^‡^ activation free enthalpy

H-bond hydrogen bond

HPLC [high performance liquid chromatography](http://www.chemguide.co.uk/analysis/chromatography/hplc.html)

GxxxG motif packing motif of two glycines separated by three amino acids

GGS glycine-glycine-serine small residue motif

GGA glycine-glycine-alanine small residue motif

*k* rate constant

Ln natural logarithm

MD molecular dynamics

Oγ third oxygen atom in the side chain

PCR polymerase chain reaction

PDB protein data bank

psaC extrinsic water soluble protein subunit of photosystem I

PSII photosystem II protein complex

PSII-RC photosystem II reaction center

PVDF polyvinylidene difluoride

*Q_A_* primary quinone

*Q_B_* secondary quinone

R^2^ squared Pearson’s correlation coefficient

RC reaction center

ΔS^‡^ activation free entropy

Sγ third sulfur element in the side chain

SDS sodium dodecyl sulfate

T temperature

ΔT temperature change

TM transmembrane

Type-II RC type II, quinon type reaction center

V volume

*V_D1-208_*  volume of amino acid residue at D1-208 site

*V_res_*  volume of amino acid residue

*wt* wild type

| PDB | Å resolution | Organism | D1-Gly207 | D1-Gly208 | D1-Ala209 | D1-Cys212 |
| --- | --- | --- | --- | --- | --- | --- |
| 3wu2 | 1.9 | *T. vulcanus* | -0.874 | -2.456 | -1.820 | -6.728 |
| 4ub6 | 1.95 (XFEL) | *T. vulcanus* | -2.262 | -3.783 | -3.119 | -8.133 |
| 4pj0 | 2.44 | *T. elongatus* | -1.464 | -2.864 | -2.209 | -7.125 |
| 3bz1 | 2.9 | *T. elongatus* | 1.479 | -3.068 | -2.431 | -7.272 |

**Supplementary Table 1: The cross-membrane depth of the investigated PSII RC residues.** The data from available high-resolution crystallography-resolved structures was aligned relative to the membrane using OPM[^1^](#_ENREF_1). In all structures, the D1-207-209 motif is within the innermost hydrocarbon region of the membrane.

| **Motif number** | **Motif** | **Organism** | **Genebank codes** |
| --- | --- | --- | --- |
| 1 | **GDC** | *Amborella trichopoda* | gi|586778932, gi|586642889 |
| 2 | **GDA** | uncultured marine virus | gi|193874354 |
| 3 | **GDS** | uncultured marine virus | gi|193874658 |
| 4 | **SAA** | *Marsilea mutica* | gi|295791991 |
| 5 | **GAS** | *Aulacoseira granulate* | gi|370991803 |
| 6 | **GSS** | *Ricinus communis* | gi|255569246, gi|255557504 |
| 7 | **GGP** | *Cornus eydeana, Limnophyton sp. 1* LYC-2011*,* uncultured bacteria (twice) | gi|325071970, gi|336445376 |
| 8 | **GGT** | uncultured cyanophage, uncultured organism, uncultured marine virus, uncultured bacterium | gi|499285757, gi|223972421, gi|193874744, gi|193874138 |
| 9 | **GGF** | uncultured | gi|499286423 |
| 10 | **GGV** | uncultured bacterium (twice) | gi|193875060, gi|193874514 |
| 11 | **GGY** | *Syzygium cumini* | gi|296936651 |
| 12 | **AGA** | *Crocosphaera watsonii* WH 8501 (5 times)*, Cyanothece* sp. PCC 8801*, Cyanothece* sp. PCC 8802 | gi|546228654, gi|494515219, gi|546218108, gi|546234468, gi|494521677, gi|501594515, gi|506265167 |
| 13 | **AGS** | *Bryum coronatum, Deutzia corymbosa, Archilejeunea planiuscula* | gi|27228029, gi|325072034m gi|119656707 |
| 14 | **SGS** | uncultured | gi|499287172, gi|193874760 |
| 15 | **DGS** | uncultured bacterium, uncultured marine organism | gi|193874694, gi|149939007 |
| 16 | **KGS** | uncultured marine virus | gi|193874982 |

**Supplementary Table 2**: **List of genes in which the D1-207-209 GGA or GGS sequence motifs are not fully conserved**. These genes make less than 0.01% of the sequence space that was analyzed. Moreover, these examples are generally of non-photosynthetic organisms, e.g. viruses, where the sequence is possibly an evolutionary fossil and is not viable.

|  |  |  |  | | |
| --- | --- | --- | --- | --- | --- |
| D1-208 Amino acid | k^ET^ [sec^-1^] | ET [%] | T_exp_ [˚K] | T_Cal_ [˚K] | 1/V_Lit_ [Å^-3^] |
| Gly | 3207.5 | 100 | 294.0 | 289 | 0.015 |
| Ala | 1945.3 | 60.6 | 271.7 | 273 | 0.011 |
| Ser | 1511.5 | 47.1 | 261.6 | 267 | 0.010 |
| Thr | 1400.8 | 43.7 | 258.7 | 254 | 0.008 |
| Gln | 805.1 | 25.1 | 239.5 | 239 | 0.006 |
| Asp | 1093.9 | 34.1 | 249.8 | 249 | 0.007 |
| Val | 1044.4 | 32.6 | 248.2 | 248 | 0.007 |

**Supplementary Table 3**: **The percentage of *Q_B_* single reduction (ET%, in isolated PSII-RC) and the ET rate (k^ET^ [sec^-1^], in whole cells) as a function of the ambient temperature and *V_res_* at D1-208 (*V_D1-208_*), respectively**. T_exp_ is the experimental temperature at which ET(%) was measured, *V_D1-208_* is the volume for which k^ET^ was measured or found by extrapolation (as explained in the text and supplementary Figure 3) and T_cal_ is the value of T calculated for *V_res_* using Eq 1. The *V_res_* values are taken from reference[^2^](#_ENREF_2).

| **D1-208 residue**  ***Q_A_^-^*🡪*Q_B_*** | **Gly (*wt*)** | **Ala** | **Ser** | **Thr** |
| --- | --- | --- | --- | --- |
| **k, s^-1^** | 2687±107 | 1945±295 | 1512±253 | 1401±193 |
| **ΔH**  [kJ•mol^-1^] | 16.76 | 17.00 | 13.53 | 15.05 |
| **TΔS**  [kJ•mol^-1^] | 36.78 | 37.16 | 41.59 | 40.48 |

**Supplementary Table 4**: **Kinetic and thermodynamics parameters for *Q_A_^-^*🡪*Q_B_* ET of the D1-Gly208 *wt* and the three viable mutants, as derived using the Eyring equation**.


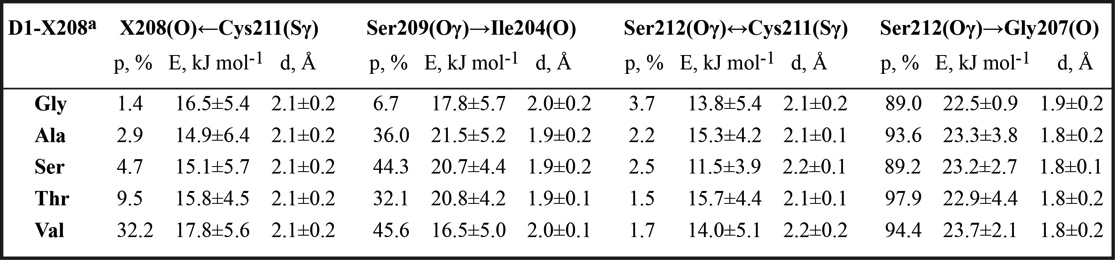


**Supplementary Table 5: Hydrogen bonds formed between *d* helices of D1 and D2 protein subunits during the 20 ns MD simulation.** The arrow points from the hydrogen bond donor towards its acceptor.

^a^ X denotes the amino acid residue present at the D1-208 site.


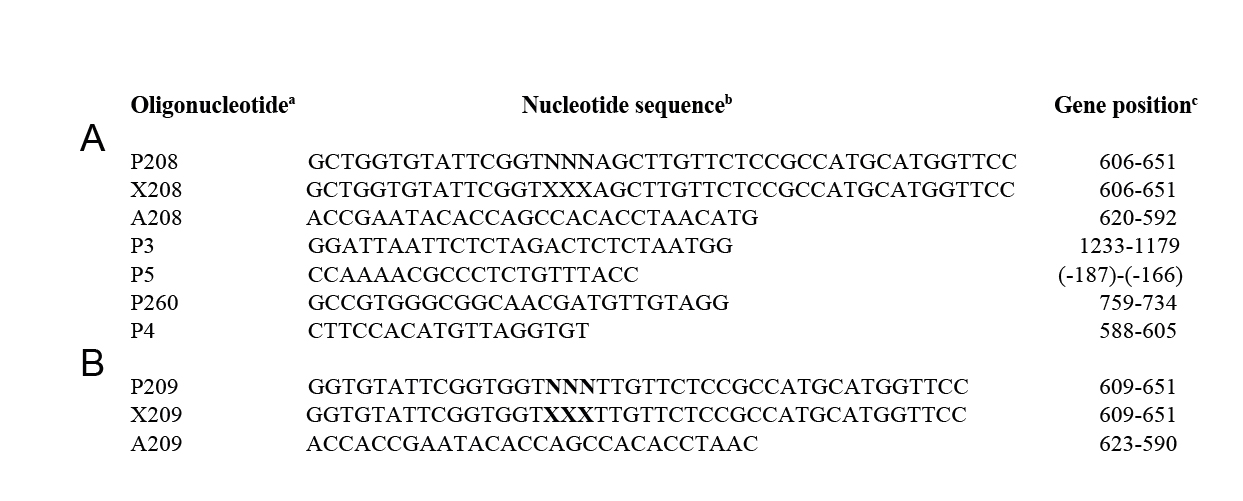


**Supplementary Table 6:** **Oligonucleotides used in combinatorial and site-directed mutagenesis.**

**(A)** Oligonucleotides used in the saturated mutagenesis of the D1-208 site. **(B)** Oligonucleotides used in the saturated mutagenesis of the D1-209 site.

^a^The use of oligonucleotides P3, P5, P208 and A208 is outlined in Supplementary Figure 1; oligonucleotides P260 and P4 were used for sequencing of the PCR product; the X208 and X209 sequence represents oligonucleotides that were used for D1-208 and D2-209 site-directed mutagenesis.

^b^The universal code for degenerate oligonucleotides. The N encodes (A, g, T, C), and XXX depicts specific codons of amino acid residues that were not obtained by NNN sequence.

^c^Oligonucleotides are listed in a 5' to 3' direction. Numbering for *psbAII* is as previously described[^3^](#_ENREF_3).

**
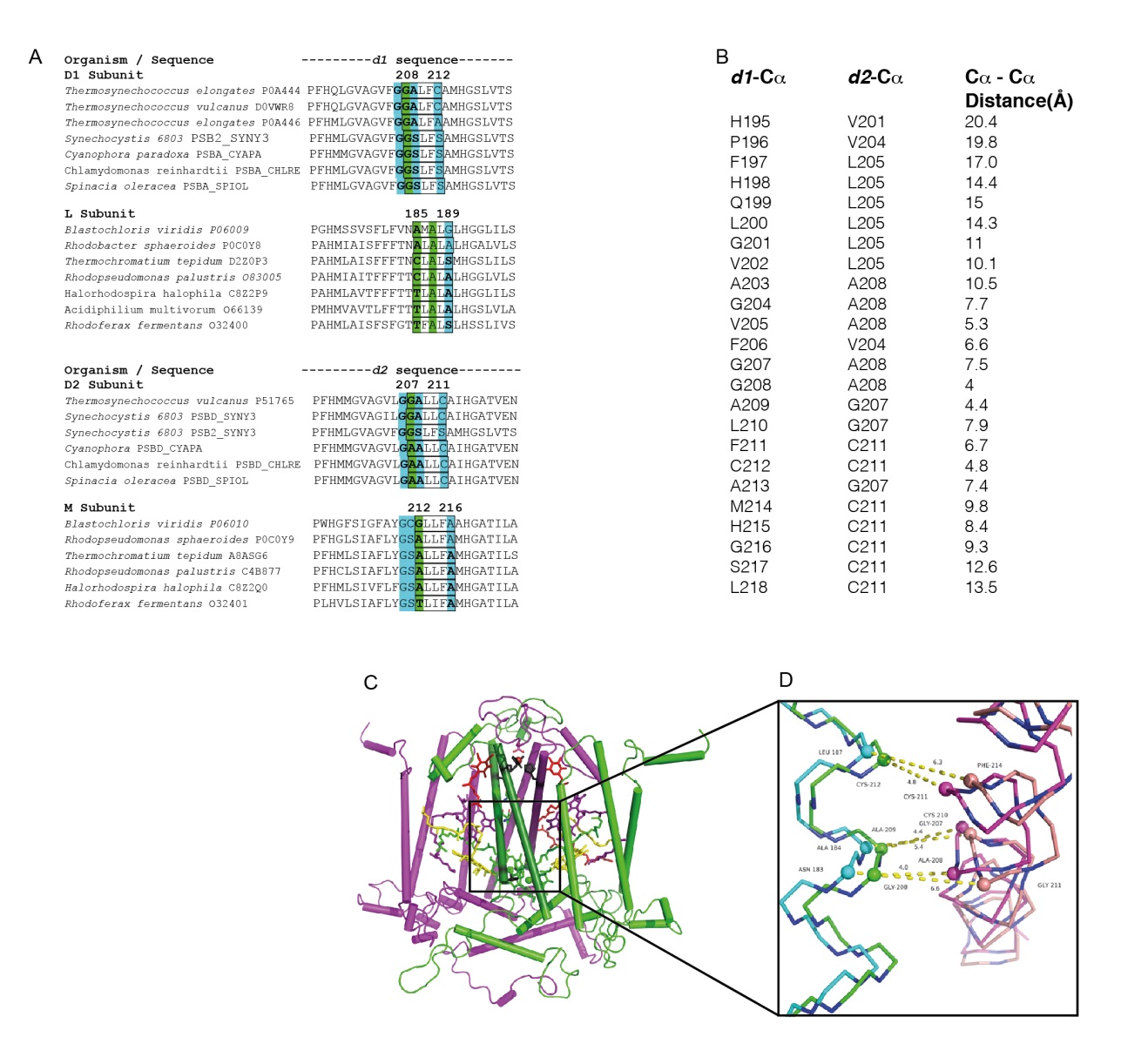
**

**Supplementary Figure 1. Sequence and structure alignment of Type-II RC *d* helices. (A)** Multiple sequence alignment of *d1* and *d2* from the pseudo-symmetric D1 and D2 subunits of representative oxygenic organisms and the L and M subunits of non-oxygenic organisms. Small amino acids in the region of interest are highlighted. A conserved G_208_xxxG_212_-like motif (boxed) is found at the center of the TM helix. Therein, the first small residue is flanked by two other small residues. The *d* helices from the bacterial RC include conserved small amino acid motifs at the center of the helix. The three consecutive small residues are conserved for the M subunit. Sequence accession numbers are taken from the UniProt database (or PDB, when available). **(B)** List of closest PSII RC intersubunit *d1-d2* Cα-Cα distances at the studied region (complementary to Fig. 1C). **(C)** The full D1-D2 10 TM helices (D1 in green and D2 in pink) with a zoom on **(D)** the structural alignment of the *d* helices from PSII (PDB: 3wu2) and bacterial RCs (*B. viridis* PDB: 1dxr, 2.0Å-resolution, depicted in cyan and light pink) and the distances between the three assessed residues.


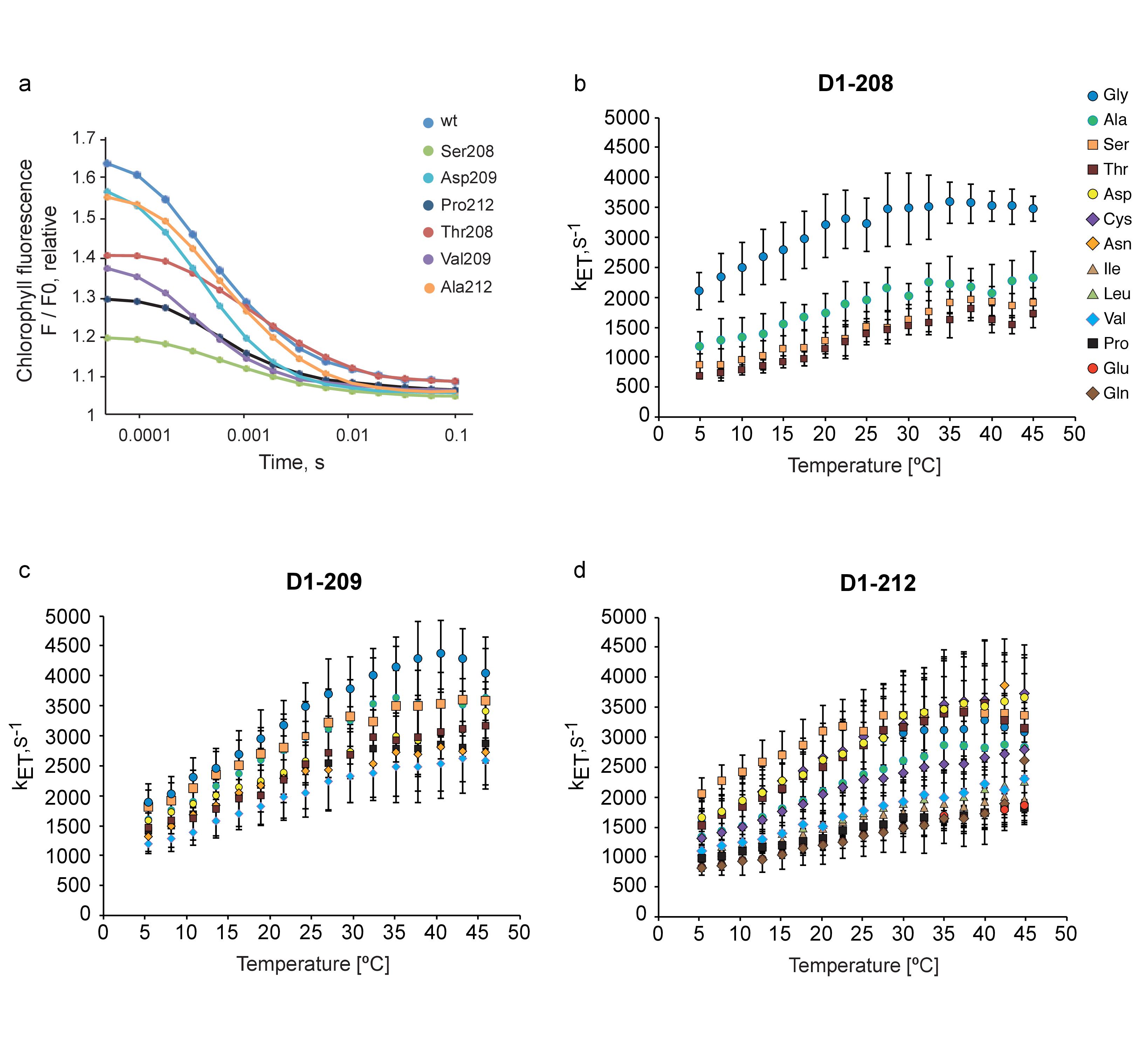
**Supplementary Figure 2: *In vivo* ET, measured by chlorophyll fluorescence decay following single turnover saturating flash**. **(A)** Examples of raw fluorescence data, for different D1 mutants, normalized to the respective Fo levels, showing the fast part of the decay up to the 100 ms following the saturating flash. Cells grown under low-intensity light were diluted to 4 µg chlorophyll mL^-1^ in fresh BG-11 medium and then dark-adapted, for 30 minutes, to completely re-oxidize the PSII. Dark adaptation proceeded on ice to counter the effect of respiration on the re-oxidation process. Chlorophyll fluorescence decay following a single-turnover saturating flash was measured by a series of short, measuring flashes (pulse duration 4 µs), at 25°C, using a FL-100 double-modulation fluorometer equipped with a TR 2000 thermoregulator (PSI Ltd., Czech Republic), as previously described . The temperature dependence of the ET rate from *Q_A_^-^* to *Q_B_* for the D1-208 mutants **(B)** and D1-209 mutants **(C)** and D1-212 mutants **(D).** The statistical analysis of temperature dependent rate constants shown as standard deviation error bars.

**
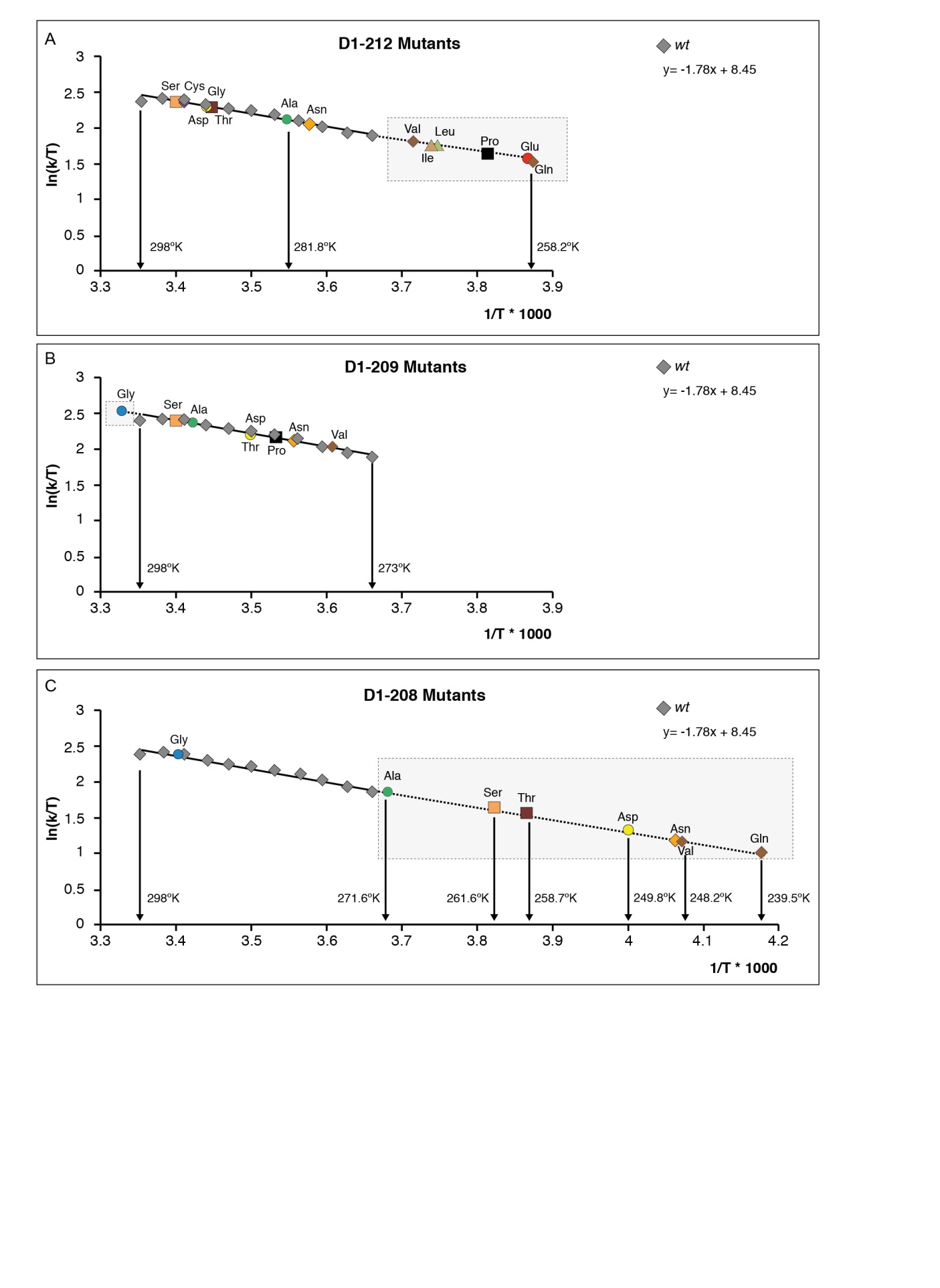
**

**Supplementary Figure 3. Translation of *V_res_* at the D1-208, D1-209 and D1-212 positions into an effective temperature for the *Q^^_A_🡪Q_B_* ET. (A)** The Ln(k/T) values measured for *wt* D1-Ser212Ser, are shown as grey diamonds against their corresponding 1/T values for the *wt* strain. The Ln(k/T) for the different residues at D1-212, is shown on the linear curve as colored symbols. The 1/T values are depicted by arrows to the 1/T axis. The D1-209 **(B)** and D1-208 **(C)** mutants are shown using a color scheme following the D1-212 mutants. Predicted 1/T values for non-viable mutants (e.g., for D1-Gly208Gln) were found by substituting *V_D1-208_* in Eq (1) for the particular residue volume. The grey box represents the extrapolated region of the WT trend-line.


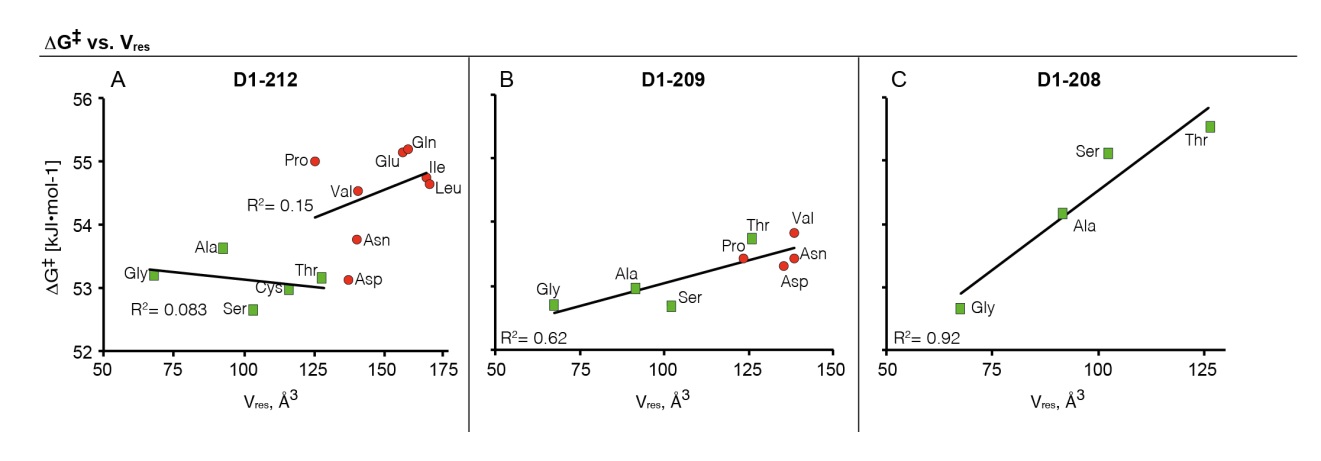


**Supplementary Figure 4. The Gibbs activation energy ΔG^‡^ plotted against V_res_ for mutations in the three studied loci. (A)** The Gibbs activation energy plotted for residues at D1-212 did not correlate with *V_res_*, while that for D1-209 **(B)** showed a mild increase with *V_res_* at a moderate correlation coefficient. In contrast, the Gibbs activation energy plotted for residues at D1-208 **(C)** steeply increased with *V_res_*, exhibiting a high correlation coefficient.


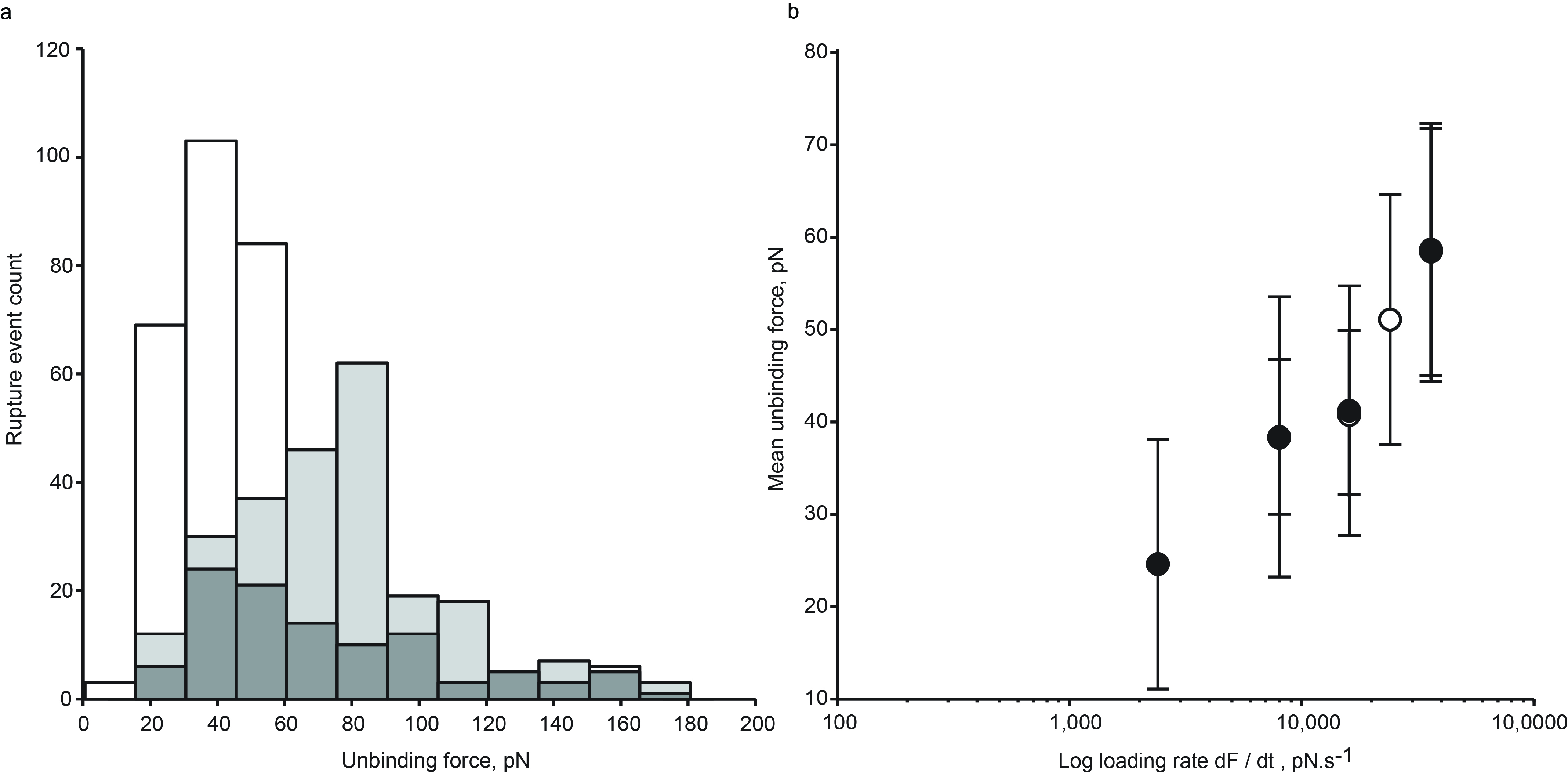


**Supplementary Figure 5. Dynamic force spectroscopy of synthetic *d1/d2* interactions.** **(A)** Rupture force distributions - Peptides measured included *d1wt* vs. *d2wt* (light gray), *d1-208Val* vs. *d2wt* (white) and control measurement (dark gray: *d1-208Val* vs. *d2wt* with *d2wt*, blocked with *d1-208Val*) recorded with sweep frequency 3Hz and binned by 15 pN. **(B)** Mean unbinding forces for *d1wt* vs. *d2wt*, performed in 0.1 % SDS (open circles, CD spectrum in Supplementary Fig. 8) and decane (gray circles). The vertical error bars represent the standard deviation values calculated from the histograms of unbinding forces binned by 5 pN.


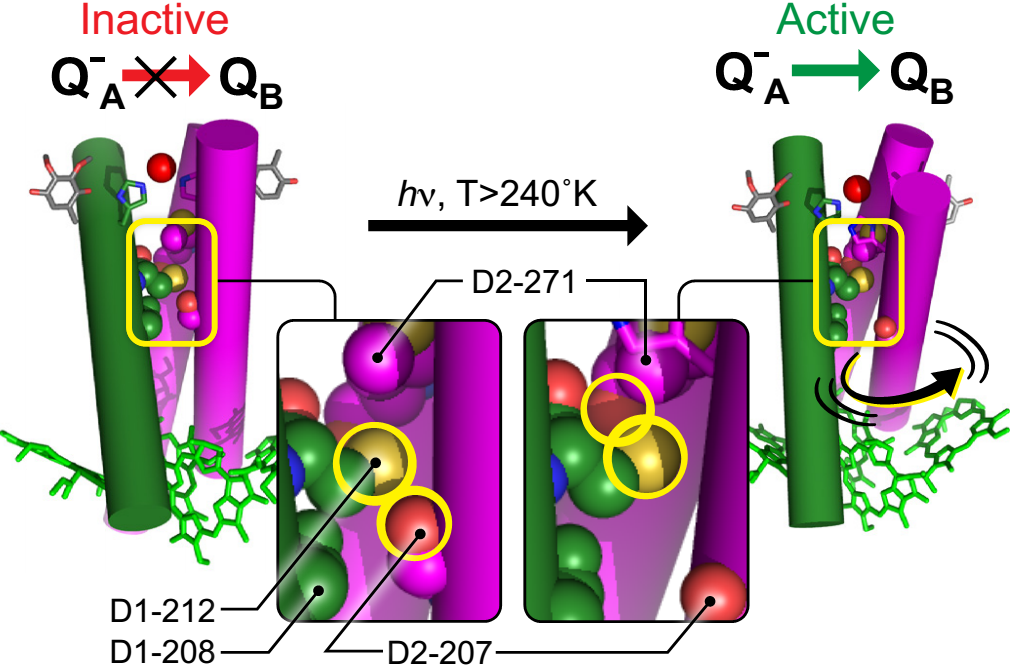


**Supplementary Figure 6. Proposed mechanism for protein-gated ET in Type II RC**. D1-208Gly as the central residue in preserving the ET-inactive conformation and opening the gate for the transient ET-active conformation. Schematically, the hydrogen bonding pattern can change in a manner dependent on D1-208: when D1-208 (denoted in green space-fill) is in close proximity to D2-207 then D1-212 can be in hydrogen bonding interactions with the backbone carbonyl of D2-207 (inactive conformation). When D2-207 is more distance, then D1-212 can accept a hydrogen bond with the backbone carbonyl of D2-271 (suggested active conformation).


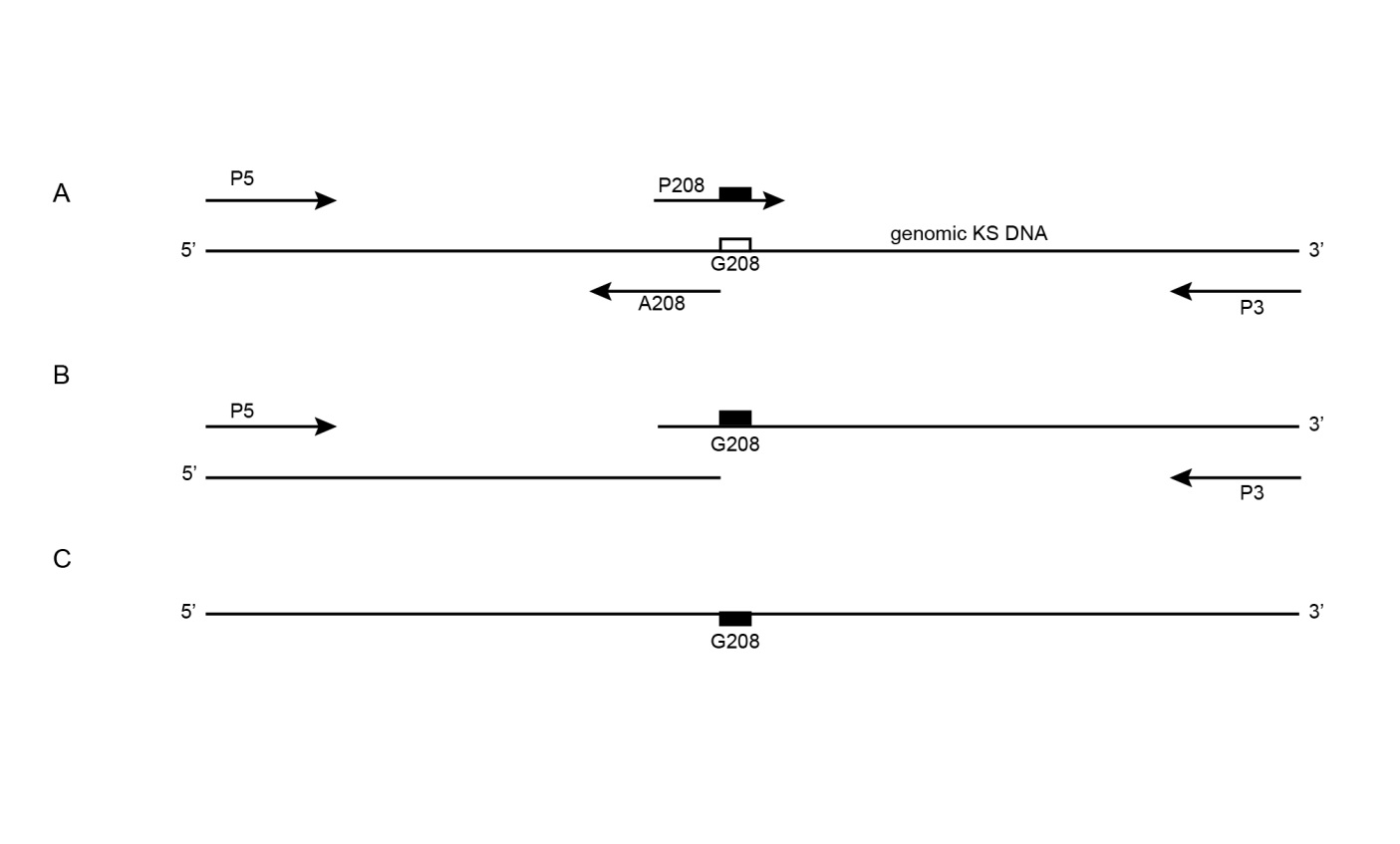


**Supplementary Figure 7. A scheme of the two-step PCR mutagenesis procedure performed to introduce a specific sequence in the KS genomic DNA (exemplified here for D1-208).** **(A)** The primers P3, P5, and A208 were designed to match known sequences in the psbAII gene (Supplementary Table 6). The primer P208 (Supplementary Table 6) contained degenerated sequence (NNN-universal code) for the D1-Gly208 codon (indicated as a black box), resulting in code for 20 amino acids. The D1-208 site in the genomic DNA is designated as an open box. **(B)** The 3’ fragment of the psbAII gene was amplified using X208 and A208 primers, resulting in a mutation near the end of the psbAII piece. The 5’ portion of the psbAII gene was amplified with P3 and P5 primers. The corresponding PCR fragments were purified from an agarose gel and mixed for a second PCR, using P3 and P5 primers. **(C)** The whole psbAII gene (1400 bp), carrying a mutation at the D1-208 site.


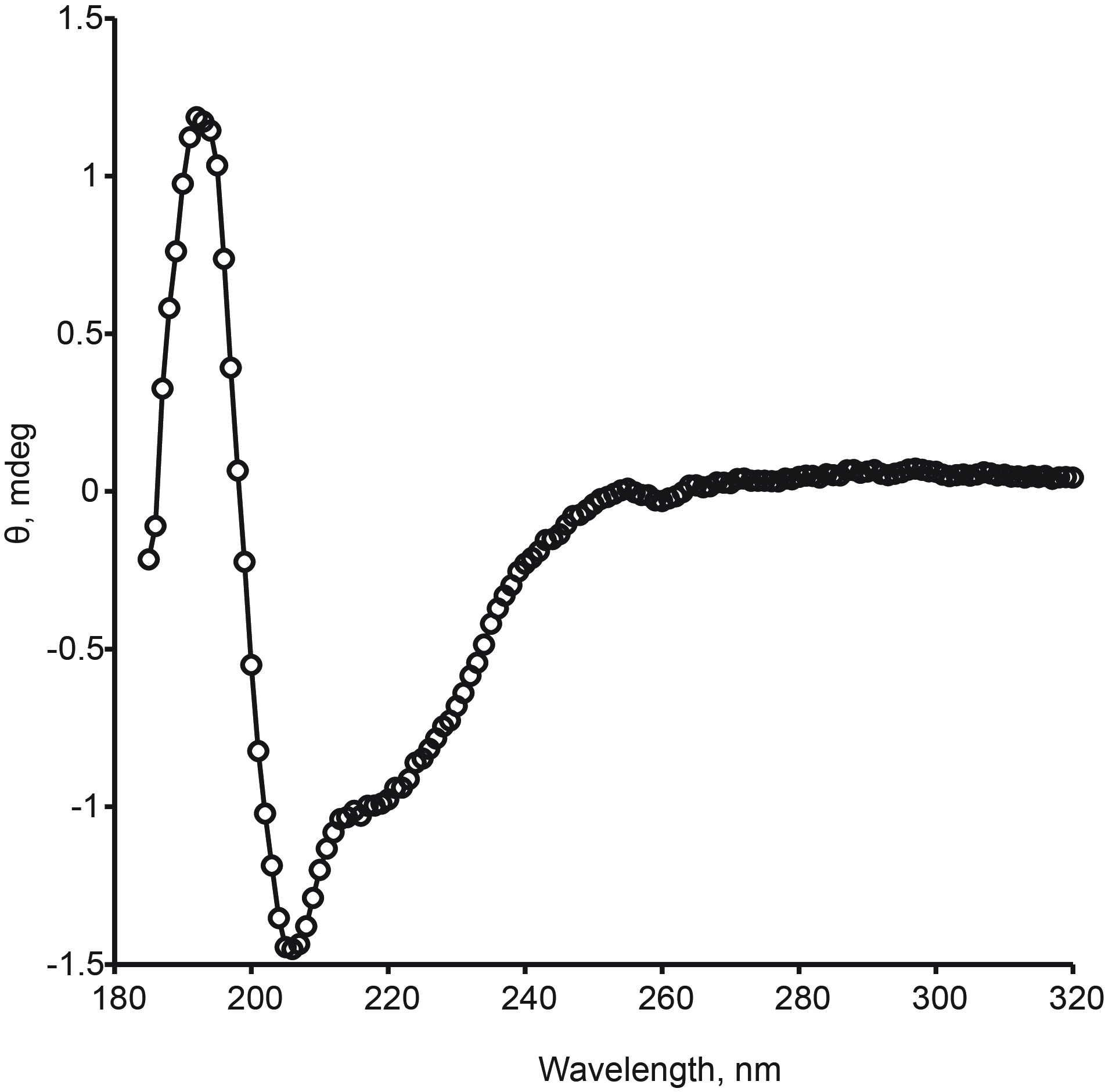


**Supplementary Figure 8. Circular dichroism spectrum of the synthesized *d1wt* peptide** (18 ng.ml^-1^) bearing the following sequence: KKPFHMLGVAGVFGGSLFSAMHGSLVTSKK; obtained in 0.1 % SDS in deionized water

**
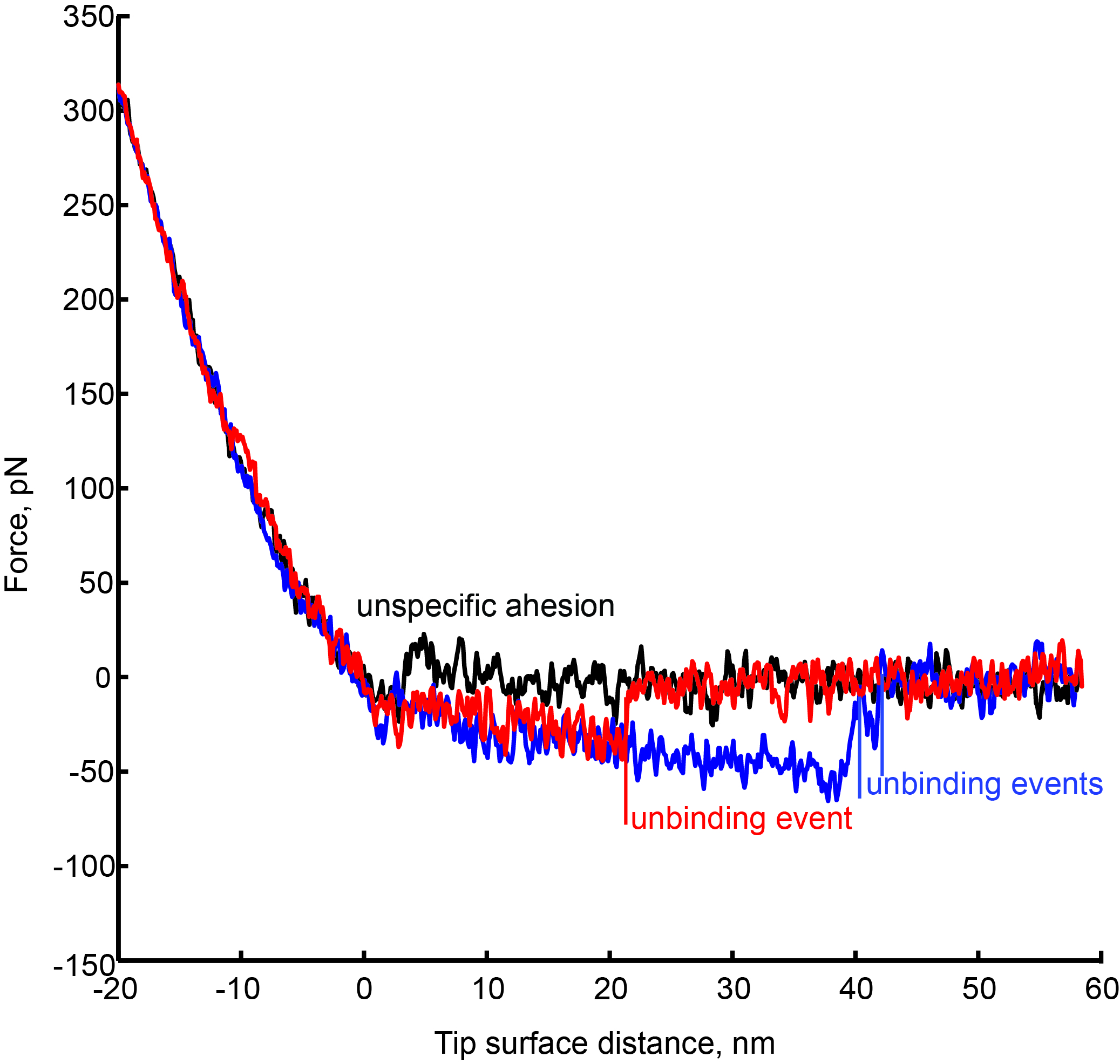
**

**Supplementary Figure 9.** **Raw retrace data of the force distance cycle.** The black line manifests the unspecific adhesion right at the tip-surface contact point. The red trace exhibits a single unbinding event corresponding to a rupture force of 29 pN. The magenta trace shows two successive unbinding events corresponding to a rupture force of 46 and 45 pN at a larger separation distance between the tip and surface. The AFM tip functionalized with *d1wt* peptide was approaching the mica functionalized with *d2wt* peptide on a vertical trajectory of 100 nm with a frequency of 10 Hz.

**References**

1. Lomize, M.A., Pogozheva, I.D., Joo, H., Mosberg, H.I. & Lomize, A.L. OPM database and PPM web server: resources for positioning of proteins in membranes. *Nucleic Acids Res* **40**, D370-376 (2012).

2. Pontius, J., Richelle, J. & Wodak, S.J. Deviations from standard atomic volumes as a quality measure for protein crystal structures. *J Mol Biol* **264**, 121-136 (1996).

3. Ravnikar, P.D., Debus, R., Sevrinck, J., Saetaert, P. & McIntosh, L. Nucleotide sequence of a second psbA gene from the unicellular cyanobacterium Synechocystis 6803. *Nucleic Acids Res* **17**, 3991 (1989).

4. Dinamarca, J. et al. Double Mutation in Photosystem II Reaction Centers and Elevated CO2 Grant Thermotolerance to Mesophilic Cyanobacterium. *Plos One* **6** (2011).

5. Shlyk-Kerner, O. et al. Protein flexibility acclimatizes photosynthetic energy conversion to the ambient temperature. *Nature* **442**, 827-830 (2006).
